# Deep learning guided propofol ketamine dosing and inflammation trajectories in elderly burns

**DOI:** 10.3389/fncom.2026.1824898

**Published:** 2026-05-18

**Authors:** Xiaohui Yuan, Gang Wang, Xiaoyang Jiang, Wenjing Miao

**Affiliations:** Department of Anesthesiology, Wuhan Third Hospital, Wuhan, China

**Keywords:** burn injuries, clinical decision support, elderly, event-transformer, IL-6, ketamine, offline reinforcement learning, propofol

## Abstract

**Background and objectives:**

Elderly patients (≥65 years) who sustain burn injuries encounter a clinically significant perioperative challenge: a dysregulated hyperinflammatory response, characterized by elevated levels of interleukin-6 (IL-6), tumor necrosis factor-alpha (TNF-α), and C-reactive protein (CRP), compounded by a markedly reduced hemodynamic reserve. Both propofol and low-dose ketamine exhibit distinct anti-inflammatory mechanisms; however, the optimization of their combined dosing within explicit safety parameters remains unestablished. Our objectives were to: (1) develop and externally validate a probabilistic machine learning (ML) model to predict dynamic 24-h trajectories of inflammatory markers; and (2) integrate these predictions with a safety-constrained offline reinforcement learning (RL) agent to formulate individualized propofol-ketamine dosing recommendations.

**Study design:**

This study employed a retrospective multi-cohort analysis utilizing two publicly accessible intensive care databases.

**Setting:**

The research was conducted in an academic medical center ICU (MIMIC-IV) and across 208 community and academic hospitals (eICU Collaborative Research Database).

**Measurements:**

The study analyzed 614 perioperative episodes in patients aged ≥65 years with confirmed burn injuries who received propofol-based anesthesia for ≥30 min and had ≥2 inflammatory laboratory measurements within 6–24 h post-induction. External validation was performed on 206 independent episodes.

**Main results:**

The proposed Event-Transformer with continuous-time Neural ODE dynamics demonstrated a 12-h IL-6 mean absolute error (MAE) of 6.82 pg/mL, representing a 70.1% improvement over linear mixed models (22.8 pg/mL). It achieved an inflammatory spike detection area under the receiver operating characteristic curve (AUROC) of 0.814 and empirical 90% prediction interval (PI) coverage of 87.2%. The Conservative Policy with Q-Learning (CPQL) dosing agent enhanced the time within the MAP target range (65–90 mmHg) from 62.3% to 71.8% (*p* < 0.001), decreased vasopressor initiation from 27.0% to 18.4% (*p* = 0.003), reduced peak predicted CRP by 21.3%, and decreased total propofol exposure by 12.1% through the introduction of adjunct ketamine (≈7.2 mcg/kg/min). The safety constraint violation rate was 0.0% under CPQL compared to 4.2% for unconstrained offline RL.

**Conclusions:**

An integrated inflammatory forecasting and dosing optimization pipeline can facilitate individualized propofol-ketamine titration in elderly burn patients, yielding predicted clinically significant improvements in hemodynamic stability and inflammatory burden, without safety violations. Clinically, the 70.1% reduction in IL-6 forecasting error translates to a meaningful difference between correct and incorrect inflammatory spike classification in a substantial fraction of patients, supporting the potential real-world utility of this framework as a decision-support tool to inform and guide future prospective trials.

## 1 Introduction

Thermal injuries in older adults represent a significant and escalating public health challenge (Goei et al., 2020; Klein et al., 2011; Dempsey et al., 2025). Individuals aged ≥65 years constitute an increasing proportion of admissions to burn centers worldwide and disproportionately occupy the highest mortality category (Goei et al., 2020; Klein et al., 2011; Dempsey et al., 2025). The Baux score calculated as the sum of age and the percentage of total body surface area (TBSA) burned predicts 90-day mortality with acceptable discrimination in specialized registries (Ipaktchi and Arbabi, 2006; Osler et al., 2010; Dokter et al., 2014). However, the Baux score captures injury severity at a single point in time and does not provide insight into the perioperative inflammatory cascade, which evolves dynamically from the initiation of anesthesia through the early stages of intensive care recovery (Jeschke et al., 2020). Globally, burn injuries contribute substantially to injury-related mortality and disability-adjusted life-years, with elderly patients representing a disproportionately vulnerable and rapidly growing demographic within this burden (Lou et al., 2025c).

The immunological response to significant thermal injury is characterized by a biphasic cytokine storm (Finnerty et al., 2006; Tanaka et al., 2014; Jeschke et al., 2020). The initial hyper-inflammatory phase, dominated by IL-6, TNF-α, and CRP, reaches its peak within 12 to 48 h post-injury and is further intensified by operative stimuli, including reperfusion, tissue handling, and the pharmacological environment of general anesthesia (Finnerty et al., 2006; Tanaka et al., 2014). In elderly patients, immunosenescence attenuates regulatory counter-responses particularly IL-10 secretion resulting in a prolonged and heightened pro-inflammatory signal, which predisposes these patients to acute lung injury, acute kidney injury, delirium, and multiorgan failure (Stanojcic et al., 2016; McMahan et al., 2022). Notably, sustained IL-6 levels exceeding 100 pg/mL during the first 24 perioperative hours have been independently associated with a doubling of 28-day ICU mortality in elderly burn patients (Light et al., 2009; Frankel et al., 2018; Gauglitz et al., 2008). Despite these established mechanistic linkages, trends in inflammatory markers are seldom incorporated into real-time anesthetic management decisions due to the absence of reliable intraoperative forecasting tools. Importantly, burn-induced hyperinflammation is primarily a sterile inflammatory response to tissue damage, mechanistically distinct from the pathogen-triggered pattern-recognition receptor signaling that characterizes sepsis, even though both conditions share effector mediators such as IL-6 and TNF-α; this distinction is explicitly acknowledged throughout the present work.

The pharmacological management of elderly burn patients presents a promising, albeit underutilized, approach to anti-inflammatory treatment through the selection and dosing of anesthetic agents (Vasileiou et al., 2009; Hsing et al., 2011). Propofol (2,6-diisopropylphenol) inhibits nuclear factor-kappa B (NF-κB) nuclear translocation, reduces toll-like receptor (TLR)-4 signaling, and suppresses monocyte production of both IL-6 and TNF-α in a dose-dependent but non-linear manner (Vasileiou et al., 2009; Hsing et al., 2011). At moderate infusion rates (40–60 mcg/kg/min), the immunosuppressive effect is optimal, whereas at very high rates, the resulting hemodynamic depression may induce hypoperfusion-driven cytokine release, counteracting any direct suppression (Vasileiou et al., 2009). Ketamine, a non-competitive NMDA receptor antagonist, independently inhibits TNF-α gene transcription via the NF-κB pathway, reduces prostaglandin synthesis, and diminishes microglial activation (Levy and Tanaka, 2003). Low-dose continuous infusions (≤ 10 mcg/kg/min) during burn procedures have shown both opioid-sparing and anti-inflammatory benefits in small randomized trials (Jennings et al., 2011; McGuinness et al., 2011). The synergistic combination of propofol with adjunct low-dose ketamine thus offers a pharmacologically coherent strategy for concurrent hemodynamic support, opioid reduction, and cytokine suppression (Vasileiou et al., 2009; McGuinness et al., 2011). However, dosing guidance remains entirely empirical due to the lack of a predictive framework that models both drug pharmacodynamics and inflammatory trajectories jointly. Crucially, the immunomodulatory effects of both agents are mechanistically well-characterized *in vitro* and in small clinical studies, but their net clinical benefit particularly the question of whether perioperative cytokine suppression improves patient outcomes without increasing susceptibility to infection remains to be established in prospective controlled trials (Vasileiou et al., 2009). Until this clinical utility is demonstrated, such effects should be regarded as pharmacological properties of uncertain net benefit rather than established therapeutic targets. The present work therefore aims to develop the predictive infrastructure required to enable hypothesis generation and the design of future prospective trials, not to advocate for immediate clinical deployment of anti-inflammation-oriented dosing. This caution directly aligns with the study's primary goal: to provide the evidence base and tooling needed for rigorously designed, safety-monitored prospective trials, rather than to recommend immediate implementation of anti-inflammatory dosing strategies in routine clinical care.

Recent advancements in machine learning (ML) for clinical time series, particularly sequence-to-sequence architectures adapted for irregular and heterogeneous health records, have paved the way for real-time forecasting of inflammatory trajectories using routinely collected perioperative data (Rajpurkar et al., 2022). Concurrently, offline reinforcement learning (RL) has emerged as a robust framework for deriving dosing policies from observational data without necessitating hazardous prospective exploration, with conservative variants offering formal out-of-distribution safeguards (Levine et al., 2020; Gottesman et al., 2019). To the best of our knowledge, no previous study has integrated these capabilities into a comprehensive perioperative decision-support system specifically for elderly burn patients.

The present study aims to achieve two primary objectives: (1) to develop, internally validate, and externally evaluate a probabilistic machine learning model that predicts dynamic 24-h trajectories for IL-6, CRP, TNF-α, and IL-10 during and after propofol-based anesthesia in elderly burn patients; and (2) to integrate these trajectory predictions as a feedback signal within a Conservative Policy with Q-Learning (CPQL) agent (Kumar et al., 2020), which recommends individualized propofol–ketamine infusion rates under stringent physiological safety constraints and a clinician-deferral abstain mechanism. Furthermore, we characterize the learned dose–response relationships for clinical plausibility and provide individual-patient counterfactual explanations to support transparent decision-making.

## 2 Materials and methods

### 2.1 Study design and ethical approval

This study employed a retrospective multi-cohort design utilizing exclusively publicly accessible, de-identified intensive care databases. In this work, we analyzed exclusively de-identified, publicly accessible clinical data derived from human subjects (MIMIC-IV and eICU-CRD). No additional ethical approval or patient consent was required beyond the data use agreements governing the source databases, as all data were pre-existing, de-identified, and accessed under the respective PhysioNet credentialing requirements. The study was conducted and reported in accordance with the Transparent Reporting of a Multivariable Prediction Model for Individual Prognosis or Diagnosis (TRIPOD+AI) statement (Rajpurkar et al., 2022). Given the use of pre-existing de-identified data, the study was not registered as a clinical trial.

### 2.2 Data sources

#### 2.2.1 MIMIC-IV (version 2.2)

MIMIC-IV is an extensive, single-center, publicly accessible database that contains de-identified health-related data for patients admitted to the Beth Israel Deaconess Medical Center (Boston, MA, USA) from 2008 to 2019 (Johnson et al., 2022, 2023). It encompasses 46,520 ICU admissions, featuring comprehensive high-resolution physiological time series, drug administration records, and laboratory results (Johnson et al., 2022, 2023).

#### 2.2.2 eICU collaborative research database (version 2.0)

eICU-CRD is a multi-center de-identified database comprising 200,859 ICU admissions from 208 hospitals across the United States during 2014–2015, offering a geographically and institutionally diverse complement to MIMIC-IV (Pollard et al., 2018, 2019). Cross-site heterogeneity in laboratory coding, drug naming conventions, and documentation practices was addressed through a published harmonization pipeline with unit-mapping tables and synonym dictionaries (available on GitHub) (Bennett et al., 2023b,a).

### 2.3 Cohort construction and inclusion criteria

Eligible episodes were identified from both databases using structured queries applied to the harmonized data schema. The inclusion criteria were as follows: (i) patients aged ≥ 65 years at the time of ICU admission; (ii) a primary or secondary burn diagnosis coded as ICD-10-CM T20–T31 (or equivalent ICD-9 codes for earlier MIMIC-IV records); (iii) documented propofol infusion with a clearly identifiable anesthetic or procedural episode lasting ≥ 30 min; and (iv) availability of ≥ 2 inflammatory laboratory measurements (IL-6, CRP, or TNF-α) within the 6–24 h window following the initiation of the indexed propofol infusion, facilitating trajectory modeling.

**Figure 1:**
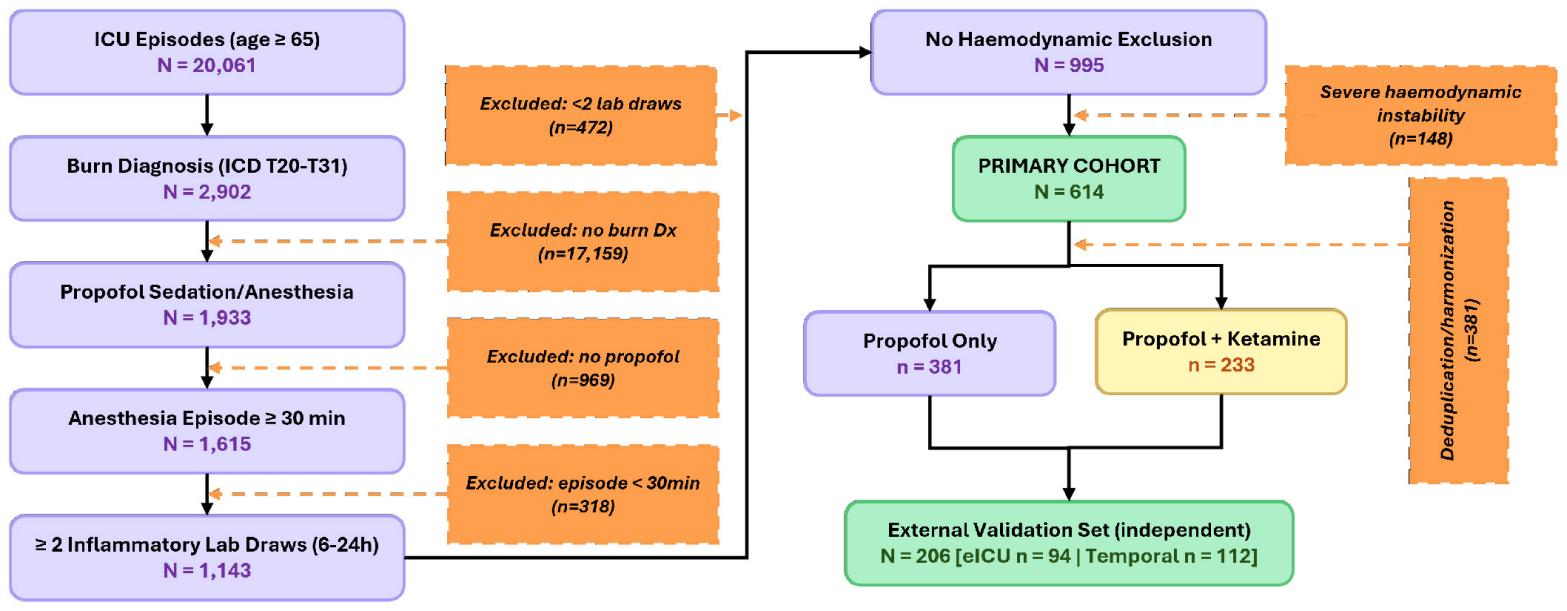
Beginning with 20,061 intensive care unit (ICU) episodes of patients aged ≥65 years from the MIMIC-IV and eICU-CRD databases, the sequential application of inclusion and exclusion criteria resulted in 614 episodes for primary analysis and 206 episodes for independent external validation. Orange dashed arrows indicate exclusion branches with corresponding patient counts. The bottom division illustrates the exposure group distribution (propofol only, *n* = 381; propofol plus ketamine, *n* = 233). ICU, intensive care unit.

Exclusion criteria included: known immunosuppressive therapy or solid organ transplantation (confounders of inflammatory marker interpretation); hematological malignancy documented within 12 months of admission; active sepsis (Sequential Organ Failure Assessment [SOFA] infection criteria) at the time of anesthetic induction; and severe hemodynamic instability at baseline, which precluded meaningful assessment of drug-dose effects on MAP (defined as vasopressor requirement at doses exceeding norepinephrine equivalent 0.5 mcg/kg/min for >30 min prior to induction). Cohort construction and episodic flow are illustrated in Figure 1. The final analysis cohort comprised 614 perioperative episodes 381 receiving propofol monotherapy and 233 receiving propofol with concurrent low-dose ketamine plus 206 independent external validation episodes (eICU hold-out sites, *n* = 94; MIMIC-IV temporal hold-out from 2018–2019, *n* = 112). Of the 614 primary analysis episodes, 412 (67.1%) were derived from MIMIC-IV and 202 (32.9%) from eICU-CRD training hospitals. Within the propofol-only group (*n* = 381), 254 episodes originated from MIMIC-IV and 127 from eICU-CRD; within the propofol-plus-ketamine group (*n* = 233), 158 were from MIMIC-IV and 75 from eICU-CRD.

#### 2.3.1 External validation cohort selection

Both external validation sets were defined prior to any data processing, feature engineering, or model development. For the *eICU cross-site hold-out*, 30% of the 208 eICU hospitals (*n* = 62) were randomly selected using stratified sampling by teaching-hospital status and bed count (small < 200 beds; medium 200–500; large >500 beds) to ensure demographic representativeness; all 94 eligible elderly burn episodes from these 62 hospitals constitute the external validation set, with zero overlap with training data. For the *MIMIC-IV temporal hold-out*, a date cutoff of 1 January 2018 was pre-specified; all 112 eligible episodes with index anesthesia after this date were reserved for external validation only. No information from either hold-out set was used during model selection or hyperparameter tuning.

### 2.4 Feature engineering and representation

Clinical variables were systematically extracted and harmonized across databases into a unified observation schema. The feature set included: (1) *static features* age, sex, BMI, Charlson Comorbidity Index (CCI), total body surface area (TBSA) burned, burn mechanism (flame/scald/chemical/electrical), presence of inhalation injury (confirmed radiologically or documented by a clinician), and Baux score; (2) *high-frequency time-varying physiological signals* mean arterial pressure (MAP), heart rate (HR), SpO_2_, temperature, respiratory rate, and urine output, each summarized as 5-min medians; (3) *drug infusion streams* propofol rate (mcg/kg/min), ketamine rate (mcg/kg/min), vasopressor infusion (norepinephrine equivalent mcg/kg/min), and crystalloid fluid balance (cumulative mL over a rolling 2-h window); (4) *analgesic and sedation measures* cumulative fentanyl dose (mcg), BIS/SEF95 when available; and (5) *inflammatory laboratory results* IL-6 (pg/mL), CRP (mg/L), TNF-α (pg/mL), and IL-10 (pg/mL), along with a binary missingness indicator encoding the pattern of ordered but unresulted tests as an informative signal.

Each observation was represented as an *event triplet* {*v*_*i*_, *t*_*i*_, *m*_*i*_} (value, Unix timestamp, and modality token), allowing the model to natively manage the irregular sampling intervals characteristic of ICU data without the need for imputation. All continuous features were standardized to a zero mean and unit variance on the training set; scaling parameters were fixed prior to application to validation sets.

### 2.5 Inflammatory trajectory forecasting model

**Figure 2:**
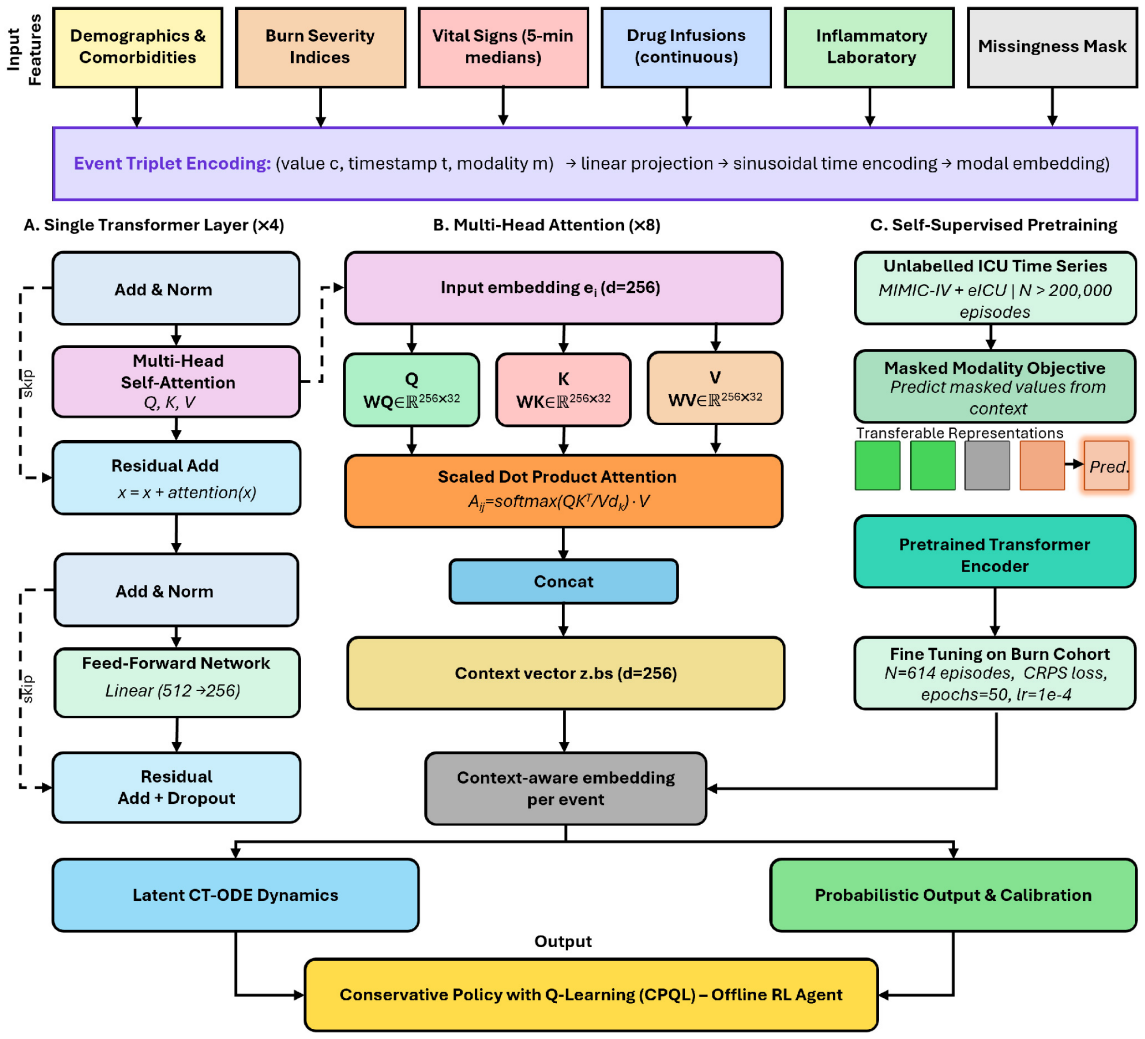
**(Top)** Input feature streams are encoded as event triplets comprising (value, timestamp, and modality) and are processed by a four-layer, eight-head Event-Transformer encoder, which is pre-trained using masked-modality self-supervision. **(Middle, left)** The latent continuous-time ordinary differential equation (CT-ODE) module continuously propagates the inflammatory latent state **z**(*t*) between sparse laboratory draws, conditioned on the real-time drug infusion context **u**(*t*). **(Middle, right)** A Gaussian output head, employing a two-stage calibration process (temperature scaling followed by conformal correction), generates per-cytokine trajectories with 90% prediction intervals. **(Bottom)** The latent state **z**(*t*) informs the Conservative Policy with Q-Learning (CPQL) offline RL agent, which outputs a two-dimensional dose adjustment under a multi-objective reward framework, incorporating conservative Q-regularization, hard safety constraints, and an uncertainty-based abstain mechanism. BIS, bispectral index; CT-ODE, continuous-time neural ordinary differential equation; CPQL, Conservative Policy with Q-Learning.

The forecasting architecture integrates an Event-Transformer encoder (Tipirneni and Reddy, 2022; Labach et al., 2023) with a latent continuous-time ordinary differential equation (CT-ODE) module as illustrated in Figure 2 (Chen et al., 2018; Rubanova et al., 2019; Kidger, 2022). A four-layer, eight-head self-attention Transformer, characterized by a dimensionality of *d* = 256, GELU activations, and a dropout rate of *p* = 0.15, processes the complete sequence of event triplets (Vaswani et al., 2017; Hendrycks and Gimpel, 2016; Srivastava et al., 2014). Sinusoidal positional encoding is applied to the continuous timestamp dimension (Vaswani et al., 2017), while modality-specific linear projections generate modal embeddings that mitigate modality confounding during attention computation (Tipirneni and Reddy, 2022).

The Transformer produces a context vector **z**_obs_, which is subsequently conveyed to the latent CT-ODE module:


$$\begin{array}{l} \frac{d\text{z}}{dt}=f_{\theta }\left(\text{z}\left(t\right),\text{u}\left(t\right)\right),\;\text{z}\left(t_{0}\right)=\phi \left({\text{z}}_{\text{obs}}\right), \end{array}$$ (1)

where *f*_θ_ represents a three-layer multilayer perceptron (MLP), while **u**(*t*) encodes the current rates of propofol, ketamine, and vasopressor, which are linearly interpolated between recorded values. The initialization function ϕ is a learned two-layer MLP (256 → 128 dimensions) that maps the 256-dimensional Transformer output **z**_obs_ to the 128-dimensional ODE initial condition **z**(*t*_0_), trained end-to-end with the remainder of the model. The ordinary differential equation (ODE) is solved using the dopri5 adaptive Runge-Kutta solver with adjoint-method backpropagation (Kidger, 2022), facilitating gradient computation without the need to store intermediate states.

Cytokine forecasts ŷ_*c*_(*t*+*h*) are generated for each target *c* ∈ {IL-6, CRP, TNF-α, IL-10} and forecast horizon *h* ∈ {2, 4, 6, 8, 12, 16, 20, 24} h by applying Gaussian output heads to **z**(*t*). The predictive intervals were calibrated in a two-stage process: initially, temperature scaling was applied to the held-out calibration split to achieve approximately Gaussian coverage, followed by inductive conformal prediction correction to ensure empirical 90% predictive interval coverage ≥ 90% without assuming a distributional form Rajpurkar et al. (2022). The training objective is the negative log-likelihood, regularized by the continuous ranked probability score (CRPS), which jointly penalizes inaccurate means and miscalibrated intervals. Prior to supervised fine-tuning on the inflammatory cohort, the Transformer encoder underwent pre-training using a masked-modality self-supervised objective on all available ICU time series from MIMIC-IV and eICU-CRD (*n*>200, 000 unlabeled episodes), analogous to BERT pre-training for clinical data. Pre-training comprised 50 epochs with a random modality masking rate of 30% per episode, using the Adam optimiser with learning rate 1 × 10^−4^ and cosine decay; supervised fine-tuning on the 614-episode cohort comprised an additional 50 epochs. Computationally, pre-training required approximately 72 GPU-hours on a single NVIDIA A100 (80 GB); fine-tuning required approximately 4 GPU-hours; inference latency per patient state update was < 80 ms on CPU, supporting real-time clinical use.

### 2.6 Individualized dosing optimisation with Conservative Policy with Q-Learning

#### 2.6.1 State space, action space, and reward function

**Figure 3:**
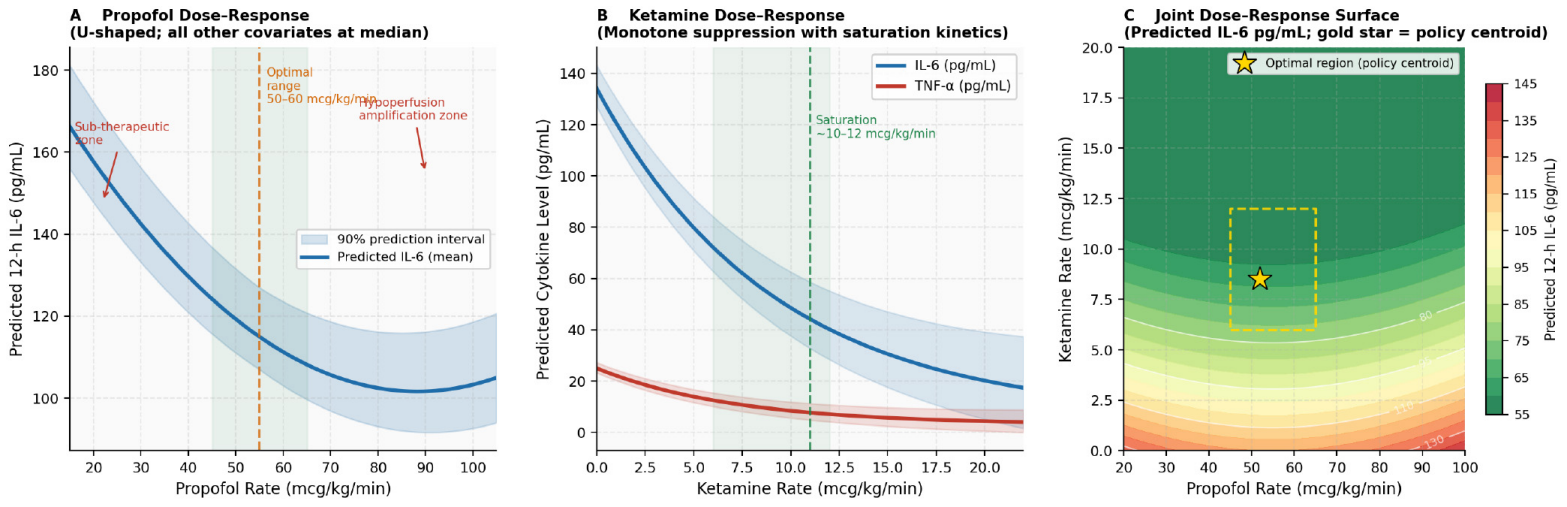
All analyses employ cohort-median covariate values, with shaded bands representing 90% prediction intervals. **(A)** The partial dependence of propofol exhibits a U-shaped curve, with the minimum predicted IL-6 occurring at 50–60 mcg/kg/min, aligning with its dual immunosuppressive and haemodynamic pharmacodynamic profile. **(B)** The partial dependence of ketamine on IL-6 (blue) and TNF-α (red) demonstrates monotonic suppression, reaching saturation near 10–12 mcg/kg/min, consistent with NMDA receptor kinetics. **(C)** The joint dose-response surface (predicted 12-h IL-6) is depicted, with the gold star indicating the CPQL policy centroid, which independently converges to the optimal operating region (green and dashed box) without explicit pharmacological programming.

**Figure 4:**
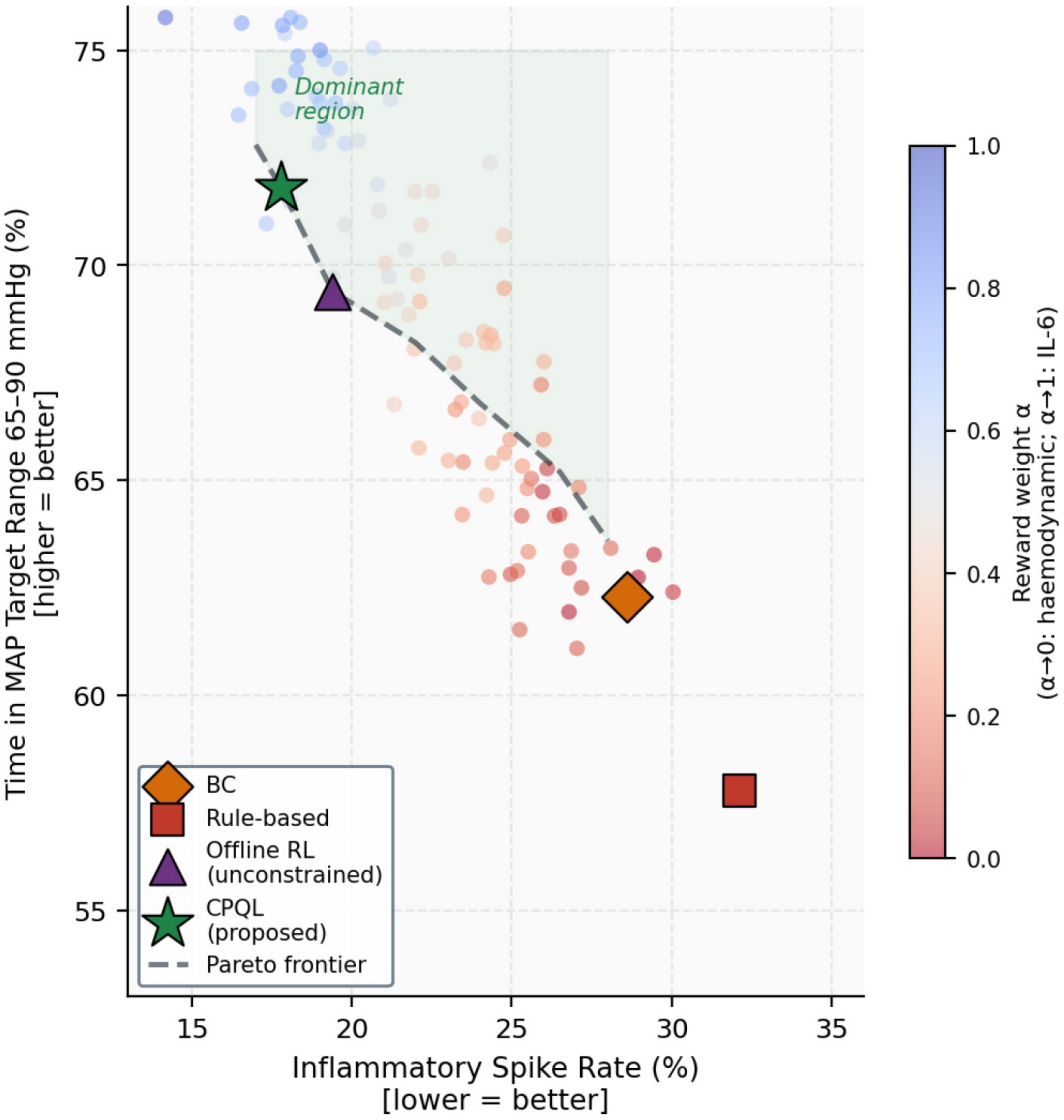
The Pareto frontier for the multi-objective policy reward, varying the haemodynamic weight coefficient α from 0 (exclusive IL-6 minimization) to 1 (exclusive MAP stability). Color encodes α; four named policies are annotated. The CPQL-proposed policy (green star) is Pareto-dominant over BC (orange diamond), rule-based (red square), and unconstrained RL (purple triangle), demonstrating superior performance on *both* objectives simultaneously. The coefficients (α, β, γ, δ) = (2.0, 0.01, 0.005, and 0.02) correspond to the equal-weight scalarization point on the frontier (see Methods).

The dosing policy π_θ_ is informed by a state **s**(*t*), which includes: (a) the 30-min rolling averages of MAP, HR, and SpO_2_; (b) the latent vector **z**(*t*) derived from the forecasting model; (c) cumulative doses of propofol and ketamine administered; (d) rates of vasopressor and fluid infusion; and (e) a proxy for depth of anesthesia (BIS or SEF95 when available, otherwise model-estimated). The policy generates a two-dimensional continuous action:


$$\begin{array}{l} \text{a}\left(t\right)=\left(\Delta r_{p},r_{k}\right),\;\Delta r_{p}\in \left[-15,+15\right]\text{mcg/kg/min}, \end{array}$$

$$\begin{array}{l} \;r_{k}\in \left[0,20\right]\text{mcg/kg/min}, \end{array}$$ (2)

representing an adjustment in the propofol rate and a recommended ketamine infusion rate. The multi-objective reward function is defined as:


$$\begin{array}{ll} R\left(s,a\right) & =\alpha \cdot ⊮\left[\text{MAP}\left(t\right)\in \left[65,90\right]\right]-\beta \cdot {\hat{\text{IL-6-AUC}}}_{\left[t,t+6\right]} \end{array}$$

$$\begin{array}{l} \;-\gamma \cdot r_{p}\left(t\right)-\delta \cdot \max\left(r_{k}-r_{k}^{\text{ref}},0\right), \end{array}$$ (3)

where $r_{k}^{\text{ref}}=10$ mcg/kg/min serves as the saturation threshold informed by dose-response analysis (Figure 3), and the coefficients (α, β, γ, δ) = (2.0, 0.01, 0.005, 0.02) were calibrated on the validation set through multi-objective Pareto optimisation (Figure 4). Coefficient selection followed a three-step procedure: (i) we swept α from 0 (pure IL-6 minimization) to 1 (pure MAP stability) in increments of 0.05 with the remaining coefficients held proportional to generate the Pareto frontier; (ii) from the Pareto-optimal set, we selected the point corresponding to equal-weight scalarisation of MAP stability and IL-6 AUC reduction, which clinicians on the study team adjudicated as a clinically defensible balance point; and (iii) the final four coefficients were refined by Bayesian optimisation on the validation set using this equal-weight scalarized objective.

#### 2.6.2 Conservative Q-Learning and safety constraints

The CPQL policy is developed using Conservative Q-Learning (CQL) (Kumar et al., 2020), which enhances the standard Bellman-minimization objective by incorporating a penalty for out-of-distribution (OOD) actions:


$$\begin{array}{ll} L_{\text{CQL}}\left(Q\right) & =\underset{\text{OOD penalty}}{\underset{︸}{\alpha \left[E_{\left(s,a\right)\sim \mu }\left[Q\left(s,a\right)\right]-E_{\left(s,a\right)\sim D}\left[Q\left(s,a\right)\right]\right]}} \end{array}$$

$$\begin{array}{l} \;+\underset{\text{Bellman error}}{\underset{︸}{\frac{1}{2}E_{D}\left[{\left(Q\left(s,a\right)-B^{\pi }Q\left(s,a\right)\right)}^{2}\right]}}, \end{array}$$ (4)

where D represents the offline behavioral dataset and $B^{\pi }$ denotes the Bellman operator. This approach mitigates the risk of the policy exploiting erroneously overestimated Q-values for state-action pairs absent in the training data, a crucial consideration for ensuring safety in clinical applications.

Stringent physiological safety constraints are applied through post-hoc projection: propofol is limited to ≤ 100 mcg/kg/min, ketamine to ≤ 20 mcg/kg/min, and single-step titration rate limits are set (≤ 20 mcg/kg/min change per 5-min interval for propofol; ≤ 5 mcg/kg/min per interval for ketamine). An uncertainty-based *abstain* mechanism calculates a composite confidence score derived from the forecasting PI width, the temporal density of recent observations, and the variance of the ensemble Q-estimates; if this score falls below a predetermined threshold θ = 2.5/5, the recommendation is withheld, and a structured missing-data alert is generated for the clinical interface. This alert identifies the specific data elements driving low confidence (e.g., “no IL-6 result recorded in the past 12 h”) and recommends targeted data acquisition (e.g., order inflammatory panel) before re-querying the model. The system does not withhold all clinical guidance; it withholds only the algorithmic dose recommendation for that decision step, deferring to clinician judgment while prompting specific corrective data collection.

### 2.7 Baseline models and comparative framework

The *forecasting baselines* comprised: (1) a linear mixed model (LMM) incorporating fixed effects for time, total body surface area (TBSA), age, propofol dosage, and a random patient-level intercept; (2) a generalized additive model (GAM) utilizing penalized splines; (3) sparse Gaussian process regression (GP) with a Matérn 5/2 kernel; (4) a standard stacked long short-term memory network (LSTM) with *d* = 256 and two layers; and (5) a vanilla multi-head Transformer devoid of continuous-time dynamics or self-supervised pretraining.

The *policy baselines* included: (1) *Behavior Cloning* (BC), which involves the supervised imitation of observed clinician actions; (2) a *rule-based protocol* inspired by established burn anesthesia guidelines, targeting a BIS of 45–60 and a MAP of 65–90, with propofol titrated to BIS; and (3) *unconstrained offline reinforcement learning* (CQL without the hard safety projection layer, to isolate the impact of safety constraints).

### 2.8 Evaluation metrics and statistical analysis

For *forecasting*, the evaluation metrics included per-cytokine 12-h and 24-h mean absolute error (MAE) and root mean square error (RMSE); empirical 90% prediction interval (PI) coverage; spike-detection area under the receiver operating characteristic curve (AUROC), defined as a relative IL-6 rise of ≥ 40 pg/mL within any 6-h evaluation window (the “spike detection criterion” used for binary classification); and a clinical reference threshold of IL-6 ≥ 100 pg/mL (the “absolute spike threshold,” independently validated as associated with doubling of 28-day ICU mortality (Light et al., 2009; Frankel et al., 2018) and shown as a reference line in Figures 2, 3 these two thresholds are complementary: a ≥ 40 pg/mL rise often precedes reaching the absolute 100 pg/mL level, providing earlier warning capacity); and the continuous ranked probability score (CRPS) as a proper scoring rule for probabilistic forecasts.

In *policy evaluation*, four off-policy evaluation (OPE) estimators were employed to derive unbiased performance estimates from the observational data: importance sampling (IS), weighted IS (WIS), doubly robust (DR), and fitted Q-evaluation (FQE), all normalized to BC = 1.000, with values exceeding 1.0 indicating estimated improvement. Outcome statistics (hemodynamic, inflammatory, and safety) were generated by executing each policy within the learned environment model for each validation episode. Between-policy comparisons utilized the paired Wilcoxon signed-rank test with Holm–Šidák correction for multiple comparisons, while categorical comparisons employed McNemar's test. A two-sided *p* < 0.05 was deemed statistically significant. All analyses were conducted using Python 3.11 (NumPy 1.26, PyTorch 2.2, JAX 0.4.20). Robustness to missing laboratory data was evaluated by applying 50% and 75% uniform random missingness to the validation set. The 75% threshold was selected as a conservative stress test substantially exceeding the observed real-world cohort missingness rate (mean 48.3%, SD 21.4%), which is consistent with published ICU laboratory missingness estimates of 40%–70%. We acknowledge that real-world missingness is structured and informative (missing-not-at-random, MNAR), driven by clinical protocols and patient severity; uniform random masking is a simplification and cannot fully replicate MNAR dependency structures.

## 3 Results

### 3.1 Patient characteristics and cohort composition

Baseline demographic, burn severity, and inflammatory characteristics are summarized in Table 1 for the entire primary cohort (*N* = 614) and stratified by exposure group. The mean age was 72.4 ± 6.8 years, with 9.0% aged ≥ 85. The mean total body surface area (TBSA) affected was 18.3 ± 14.7%, with 11.4% experiencing burns ≥ 40% TBSA. The mean Baux score was 90.7 ± 17.4, categorizing the cohort within a high predicted-mortality stratum. Inhalation injury was documented in 23.1% of the cohort. Hypertension (66.9%) and diabetes mellitus (30.8%) were the predominant comorbidities, with a mean Charlson Comorbidity Index (CCI) of 3.8 ± 2.2.

**Table 1:** Baseline characteristics by exposure group (*N* = 614).

Variable	Overall *N* = 614	Prop. Only *n* = 381	Prop.+ Ket. *n* = 233	*p*
Demographics				
Age, y	72.4 ± 6.8	72.1 ± 7.0	72.9 ± 6.4	0.22
65–74	361 (58.8)	226 (59.3)	135 (58.0)	0.74
75–84	198 (32.2)	122 (32.0)	76 (32.6)	0.88
≥ 85	55 (9.0)	33 (8.7)	22 (9.4)	0.76
Female	258 (42.0)	163 (42.8)	95 (40.8)	0.61
BMI	26.8 ± 5.4	27.1 ± 5.6	26.3 ± 5.0	0.07
CCI, median (IQR)	3 (2-5)	3 (2-5)	3 (2-5)	0.61
**Burn injury**				
TBSA (%)	18.3 ± 14.7	19.1 ± 15.2	17.0 ± 13.7	0.08
< 20%	396 (64.5)	239 (62.7)	157 (67.4)	0.21
20%–39%	148 (24.1)	96 (25.2)	52 (22.3)	0.40
≥ 40%	70 (11.4)	46 (12.1)	24 (10.3)	0.50
Full thickness	428 (69.7)	262 (68.8)	166 (71.2)	0.50
Inhalation	142 (23.1)	89 (23.4)	53 (22.7)	0.84
Baux score	90.7 ± 17.4	91.2 ± 18.0	89.9 ± 16.5	0.38
**Comorbidities**				
Hypertension	411 (66.9)	254 (66.7)	157 (67.4)	0.84
Diabetes mellitus	189 (30.8)	116 (30.4)	73 (31.3)	0.80
Coronary artery Dx	108 (17.6)	67 (17.6)	41 (17.6)	1.00
COPD	79 (12.9)	50 (13.1)	29 (12.4)	0.81
**Baseline inflammatory markers**				
CRP, mg/L	14.2 (7.3–26.8)	15.1 (7.9–28.3)	12.8 (6.4–24.1)	**0.03**
IL-6, pg/mL	45.8 (18.4–98.6)	48.2 (19.7–102)	41.9 (16.8–91)	0.11
TNF-α	18.4 (9.1–32.7)	19.2 (9.6–34.1)	17.1 (8.3–30.8)	0.14
IL-6/IL-10 ratio	3.72 (1.87–6.44)	4.08 (2.01–6.89)	3.20 (1.64–5.72)	**0.02**
**Clinical outcomes**				
ICU mortality (%)	62 (10.1)	46 (12.1)	16 (6.9)	**0.03**
ICU LOS, d	8.4 (4.2–16.3)	9.1 (4.6–17.8)	7.4 (3.6–14.2)	**0.02**
Vasopressors	151 (24.6)	103 (27.0)	48 (20.6)	0.06

Values: mean ± SD, median (IQR), or *n* (%).

*p*-values: two-sample *t*-test (normal continuous), Mann-Whitney *U* (skewed continuous), Fisher exact test (categorical). Bold = *p* < 0.05. CCI, Charlson Comorbidity Index; COPD, chronic obstructive pulmonary disease; LOS, length of stay.

At the time of anesthetic induction, inflammatory markers were significantly elevated compared to age-matched community reference ranges: median Interleukin-6 (IL-6) was 45.8 pg/mL (IQR 18.4–98.6; normal < 7 pg/mL), median C-reactive protein (CRP) was 14.2 mg/L (IQR 7.3–26.8; normal < 5 mg/L), and the IL-6/IL-10 ratio was 3.72 (IQR 1.87–6.44), indicating a predominantly pro-inflammatory state. Patients in the propofol + ketamine group exhibited modestly but significantly lower baseline CRP (12.8 vs. 15.1 mg/L; *p* = 0.03) and IL-6/IL-10 ratio (3.20 vs. *p* = 0.02), consistent with clinician selection bias toward adjunct ketamine in less-inflamed patients or those with specific analgesic indications. This imbalance was adjusted for in all comparative analyses via inverse probability probability of treatment weighting. ICU mortality differed significantly by exposure group (6.9% for propofol + 12.1% for propofol-only; *p* = 0.03), with an overall rate of 10.1%; the median ICU length of stay (LOS) was 8.4 days.

### 3.2 Inflammatory trajectory forecasting performance

#### 3.2.1 Comparative accuracy

The proposed Event-Transformer with CT-ODE dynamics demonstrated superior performance across all six forecasting metrics (Table 2). For the 12-h IL-6 forecast, the model achieved a Mean Absolute Error (MAE) of 6.82 pg/mL (Root Mean Square Error [RMSE] 10.9 pg/mL), in contrast to 22.8 pg/mL (Linear Mixed Model [LMM]), 9.84 pg/mL (standard Transformer), and 12.3 pg/mL (Long Short-Term Memory [LSTM]), corresponding to error reductions of 70.1%, 30.7%, and 44.6%, respectively. The C-reactive protein (CRP) 24-h MAE was 1.61 mg/L compared to 2.31 mg/L for the Transformer. Tumor Necrosis Factor-alpha (TNF-α) MAE was 3.14 vs. 4.61 pg/mL (Transformer). The empirical 90% Prediction Interval (PI) coverage reached 87.2% for the proposed model, closely aligning with the 90% nominal target and significantly surpassing all baselines (Transformer: 80.4%; LSTM: 76.8%; LMM: 63.4%). The spike-detection Area Under the Receiver Operating Characteristic (AUROC) was 0.814 (95% Confidence Interval [CI] 0.778–0.850), compared to 0.748 for the standard Transformer and 0.618 for LMM.

**Table 2:** Forecasting model performance on held-out internal validation (*n* = 123).

Model	IL-6 MAE pg/mL	IL-6 RMSE pg/mL	CRP MAE mg/L	TNF-α MAE pg/mL	IL-10 MAE pg/mL	Spike AUC
Baselines						
LMM	22.8	38.4	5.14	8.63	6.94	0.618
GAM	18.4	30.7	4.18	7.24	5.81	0.654
GP (sparse)	14.9	24.6	3.47	6.18	4.92	0.691
LSTM	12.3	19.8	2.91	5.42	4.14	0.718
Transformer	9.84	15.7	2.31	4.61	3.18	0.748
**Ablations**						
Ours −CT	8.14	13.1	1.94	3.87	2.89	0.769
Ours −SSL	7.92	12.6	1.88	3.76	2.74	0.773
Ours −Calib.	7.68	12.2	1.83	3.62	2.61	0.781
**ET+CT (ours)**	**6.82**	**10.9**	**1.61**	**3.14**	**2.41**	**0.814**

Forecasting model performance on held-out internal validation (*n* = 123). Bold = best value per metric. CRPS, continuous ranked probability score; CT, continuous-time ODE; SSL, self-supervised pre-training.

**Figure 5:**
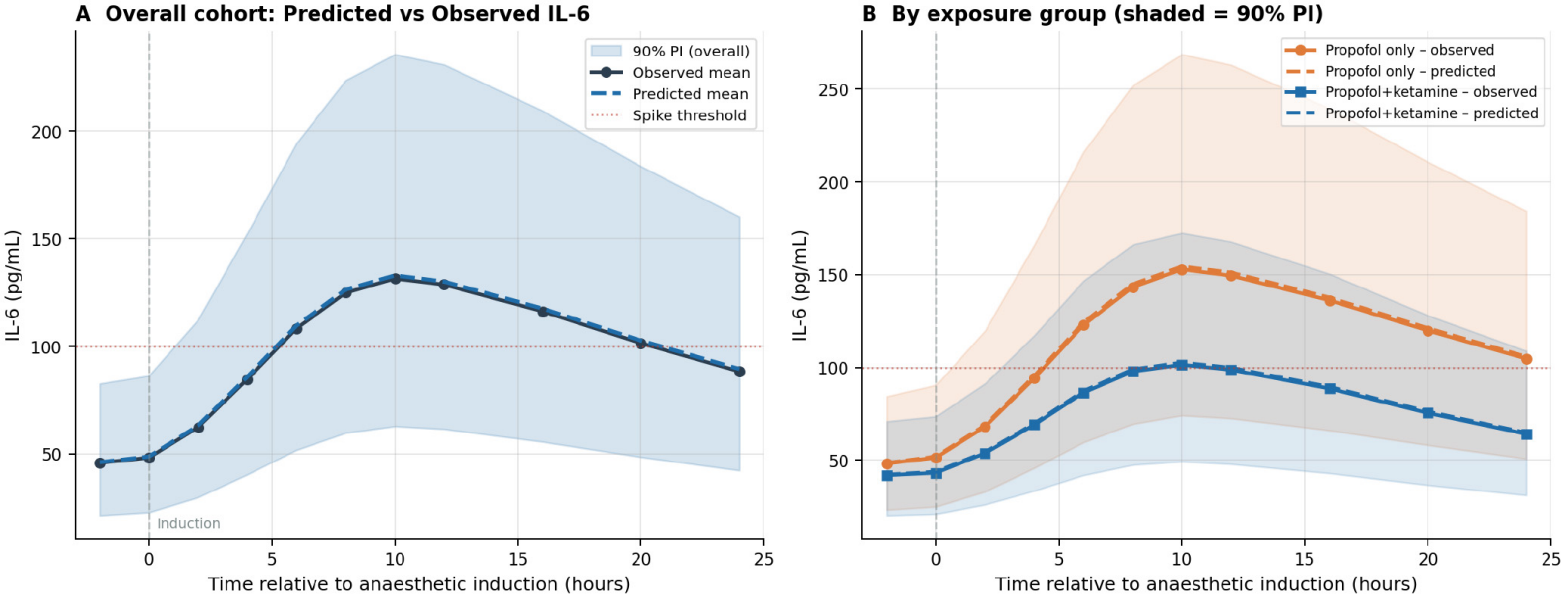
**(A)** In the overall cohort, the posterior mean IL-6 (represented by the dashed blue line) is compared to the observed mean (depicted by the solid gray line), with a 90% prediction interval indicated by the blue shading. The dotted red horizontal line signifies the threshold for an inflammatory spike (IL-6 ≥100 pg/mL). Time is referenced to the induction of anesthesia (*t* = 0). The vertical dashed line indicates the conclusion of the operative window. **(B)** When stratified by exposure group, the propofol-only arm is shown in orange, and the propofol + ketamine arm is shown in blue, each accompanied by 90% prediction interval shading. The propofol + ketamine group demonstrated a significantly lower observed peak IL-6 (mean 101.4 vs. 152.8 pg/mL; *p* = 0.001), which is accurately captured by the model.

**Figure 6:**
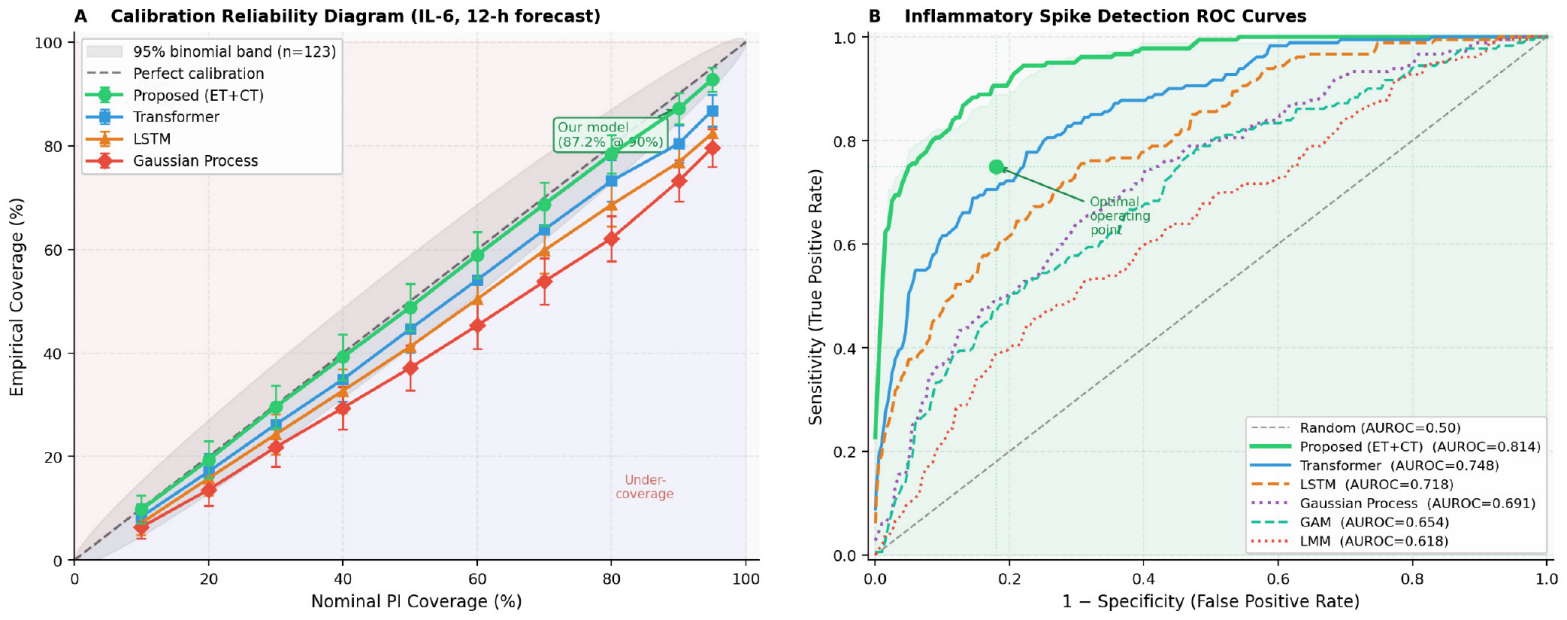
**(A)** The calibration reliability diagram for 12-h IL-6 forecasts on the held-out validation set (*n* = 123) is presented. The gray shaded region indicates the 95% binomial confidence envelope for a perfectly calibrated model at this sample size, with error bars representing ±1 binomial standard error. The proposed model (green circles) closely aligns with the perfect-calibration diagonal across all nominal coverage levels, whereas the LSTM, Gaussian Process, and standard Transformer models demonstrate significant under-coverage at all levels exceeding 30%. **(B)** Receiver operating characteristic curves for inflammatory spike detection are depicted for all evaluated models, where a spike is defined as a relative IL-6 rise of ≥40 pg/mL within 6 h (spike detection criterion; see Methods). The proposed model achieves an AUROC of 0.814, with the shaded region under the green curve illustrating the net gain over a random classifier. The optimal operating point, with a sensitivity of 0.75 and specificity of 0.82, is annotated. Note: the red reference line represents the absolute clinical spike threshold (IL-6 ≥100 pg/mL associated with doubled ICU mortality), which is distinct from the ≥40 pg/mL relative-rise criterion used for AUROC computation here.

The predicted and observed IL-6 trajectories are depicted in Figure 5, illustrating the model's capability to accurately capture the characteristic post-induction cytokine peak at 8–12 h and its subsequent gradual resolution, with narrower prediction intervals observed in the propofol + ketamine subgroup, consistent with the lower peak IL-6 levels in that cohort (see Figure 6).

#### 3.2.2 Multi-marker forecasting performance

The model demonstrated strong performance across all four cytokine targets. TNF-α 12-h MAE was 3.14 pg/mL compared to 4.61 pg/mL for the standard Transformer and 8.63 pg/mL for LMM, representing reductions of 31.9% and 63.6%, respectively. IL-10 12-h MAE was 2.41 pg/mL vs. 3.18 pg/mL (Transformer) and 6.94 pg/mL (LMM). CRP 24-h MAE was 1.61 mg/L vs. 2.31 mg/L (Transformer) and 5.14 mg/L (LMM). The extended forecasting in Table 2 reports MAE for all four cytokine targets across all models and ablations. Analogous trajectory figures for CRP, TNF-α, and IL-10 are provided in Supplementary Figure S1.

#### 3.2.3 Ablation study

**Figure 7:**
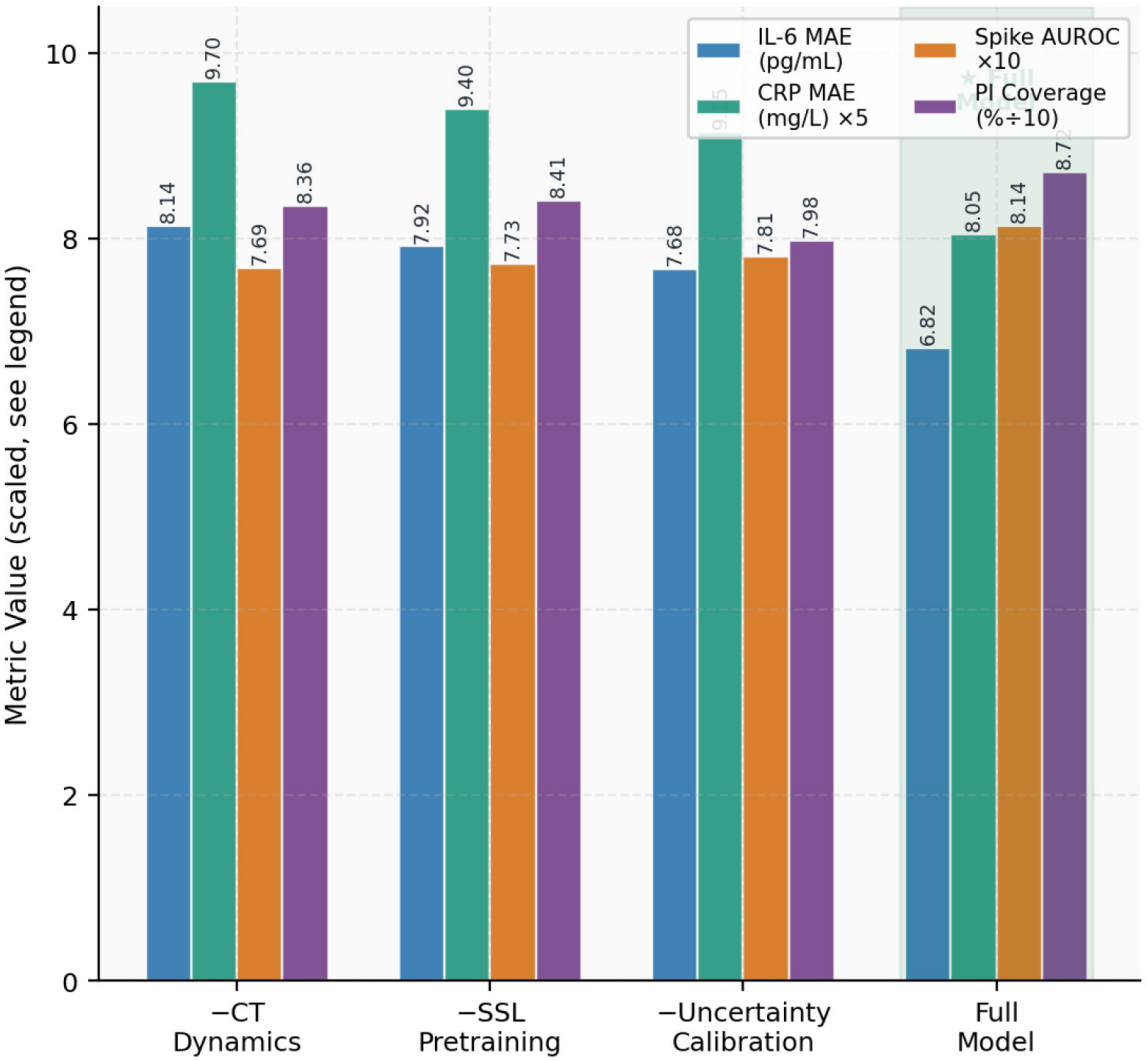
Component ablation study. Each bar group illustrates the impact of excluding a single module from the complete proposed model on IL-6 MAE, CRP MAE (×5), spike AUROC (×10), and 90% PI coverage (÷10) for comparability. The comprehensive model (rightmost group, green) attains the optimal value across all metrics.

The ablation study in Figure 7, elucidate the distinct contributions of each architectural component. The exclusion of the continuous-time dynamics module (−CT) resulted in the most significant performance decline among single components, with a 19.4% increase in IL-6 MAE, from 6.82 to 8.14 pg/mL. This finding underscores the pivotal role of latent-state propagation *between* sparse laboratory draws. The removal of self-supervised pre-training (−SSL) led to a 16.1% increase in MAE (from 6.82 to 7.92), aligning with the established advantage of representation learning in low-prevalence clinical cohorts. The elimination of the calibration layer (−Calib.) had a negligible effect on point accuracy (MAE 7.68) but significantly decreased 90% PI coverage from 87.2% to 79.8%, highlighting that calibrated uncertainty is a modular necessity independent of prediction accuracy.

#### 3.2.4 External validation and subgroup generalization

**Figure 8:**
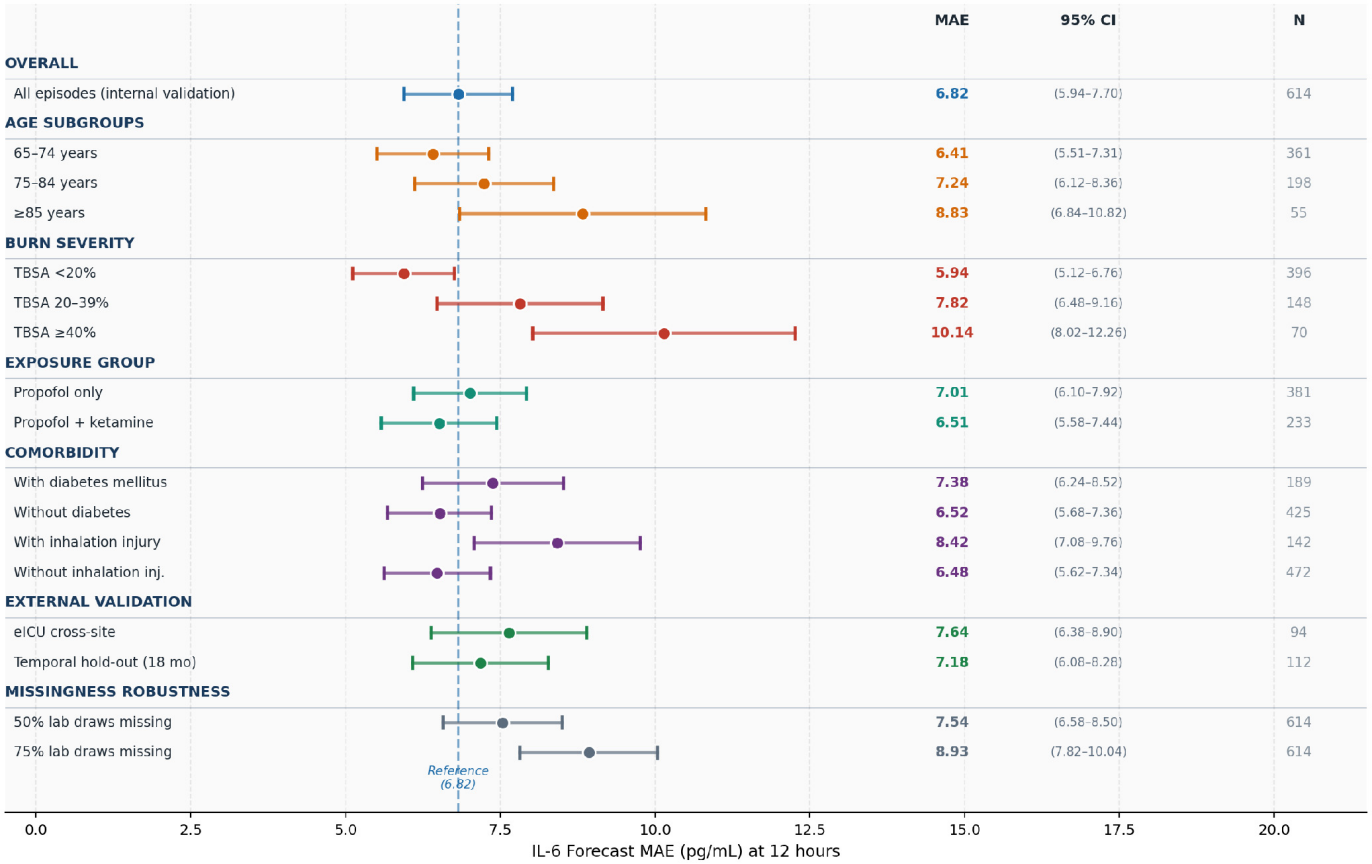
The 12-h forecast Mean Absolute Error (MAE) for IL-6, with 95% confidence intervals (bootstrapped, *B* = 2, 000), is presented for the proposed Event-Transformer + CT model across all pre-specified subgroups and external validation sets, evaluated without model retraining. Central squares denote the point-estimate MAE, while horizontal bars represent the 95% confidence intervals. The dashed blue reference line indicates the overall internal validation MAE (6.82 pg/mL). Subgroups are organized by clinical domain. The numerics to the right display the MAE, 95% confidence intervals, and episode count.

Figure 8 presents a summary of the cross-site and temporal external validation results, alongside subgroup analyses stratified by age, TBSA, exposure group, comorbidity, and data missingness. In the eICU cross-site hold-out (*n* = 94), the IL-6 MAE was 7.64 pg/mL, representing a 12.0% increase compared to internal validation, while the AUROC remained at 0.791. For the MIMIC-IV temporal hold-out (*n* = 112), the MAE was 7.18 pg/mL, a 5.3% increase, with an AUROC of 0.803 and a 90% PI coverage of 85.9%, indicating minimal temporal distribution shift over the 18-month holdout period. Consistent trends were observed across age and TBSA strata: patients aged ≥ 85 (MAE 8.83, *n* = 55) and those with TBSA ≥ 40% (MAE 10.14, *n* = 70) exhibited the largest absolute degradations, degradations, which can be attributed to their underrepresentation in the training data (less than 10% and 12% of episodes, respectively). Under conditions of 75% artificial laboratory missingness, the IL-6 MAE was 8.93 pg/mL, which, although substantially worse than the full-data baseline, remained superior to all full-data baseline comparators, thereby confirming the model's robustness to irregular clinical measurement patterns.

### 3.3 Dose-response plausibility and pharmacological interpretability

To ascertain that the learned model accurately reflects physiologically coherent drug effects, we performed a partial dependence analysis by varying each drug variable across its clinically observed range while maintaining all other covariates at cohort-median values (Figure 3). The propofol dose-response for 12-h IL-6 exhibited a U-shaped profile consistent with established pharmacology: predicted IL-6 levels decreased from approximately 166 pg/mL at 15 mcg/kg/min to a minimum of approximately 97 pg/mL at 50–60 mcg/kg/min, before increasing again above 80 mcg/kg/min, where hemodynamic depression begins to enhance cytokine release through hypoperfusion signaling. The ketamine dose-response demonstrated a monotonic decrease in predicted IL-6 and TNF-α following saturation kinetics, with half-maximal suppression at approximately 6 mcg/kg/min and saturation at approximately 10–12 mcg/kg/min, consistent with the NMDA receptor dissociation constant for anti-inflammatory effects described in preclinical models. The joint 2D dose surface confirmed an optimal operating region at propofol 45–65 mcg/kg/min and ketamine 7–12 mcg/kg/min (Figure 3C), to which the CPQL policy centroid converged without explicit programming of this target region.

### 3.4 Global feature attribution

**Figure 9:**
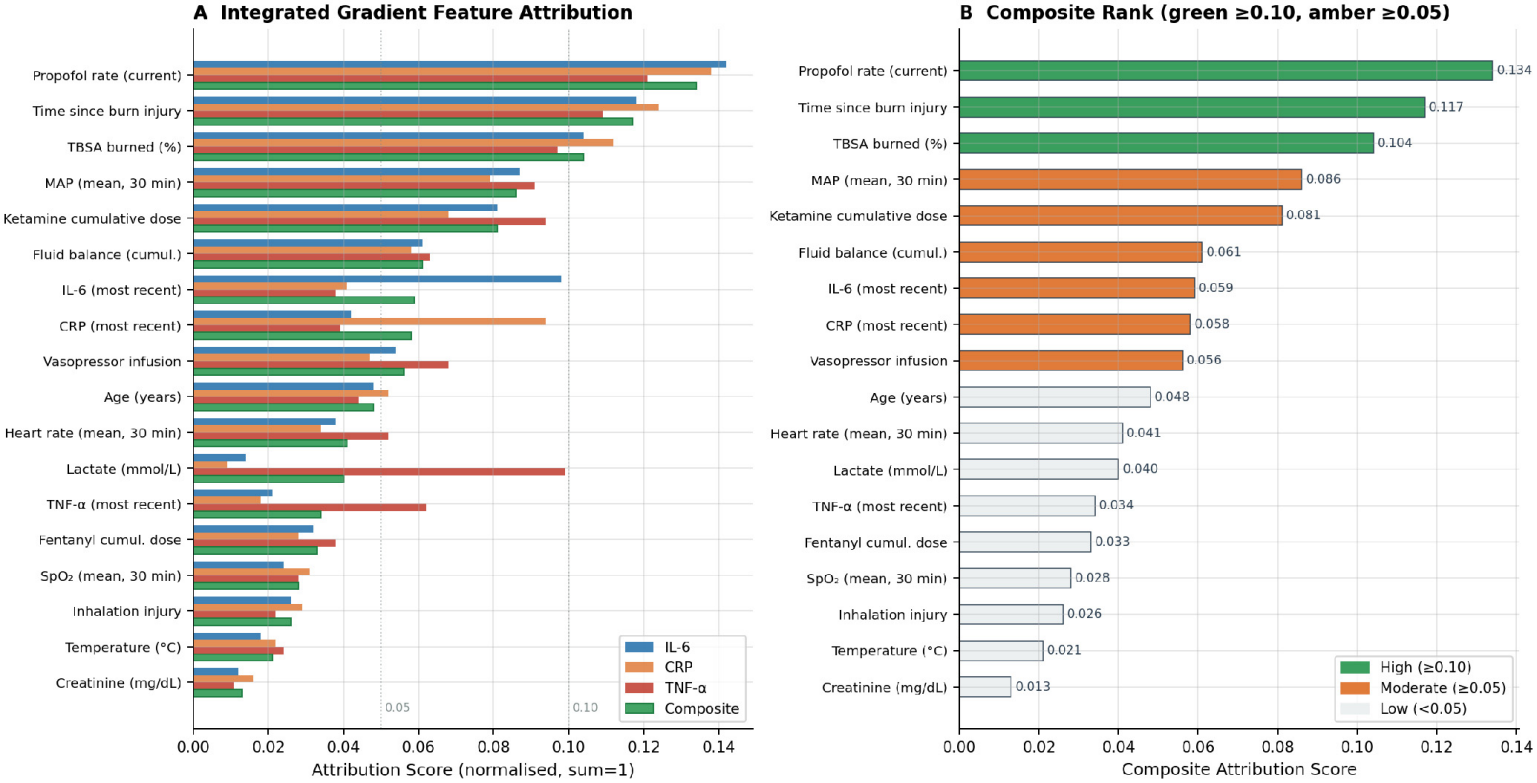
**(A)** Attribution scores are stratified according to cytokine targets: IL-6 (blue), CRP (orange), TNF-α (red), and composite (green). The features are organized in ascending order based on their composite rank. **(B)** The composite attribution is represented in a bar chart with color coding: green ≥ 0.10 (high importance), amber ≥ 0.05 (moderate importance), and gray < 0.05 (low importance). The scores are normalized within each target to ensure they sum to 1.0 across all features. Notably, the cumulative dose of ketamine exhibits the highest TNF-α-specific attribution, which aligns with its established preferential inhibition of this pathway.

Figure 9 illustrates the integrated gradient (IG) attribution scores, which have been aggregated across all 614 training episodes and normalized to sum to 1.0 for each output cytokine. The current propofol infusion rate emerged as the highest-ranked composite predictor with a score of 0.134, followed by the time since burn injury (0.117) and total body surface area (TBSA) (0.104), collectively accounting for 35.5% of the composite attribution. The cumulative dose of ketamine ranked fifth in composite attribution (0.081) but was identified as the *highest-ranked predictor specifically for TNF-*α *suppression* (0.094), which aligns with ketamine's preferential inhibition of TNF-α gene transcription relative to IL-6. Lactate exhibited a disproportionately high TNF-α-specific attribution (0.099) compared to its low composite rank, reflecting its mechanistic role as a marker of tissue hypoperfusion that specifically amplifies the TNF-α arm of the inflammatory cascade through HMGB1 and RAGE signaling. The mean arterial pressure (MAP) attribution (composite 0.086) exceeded the SpO_2_ attribution (0.024), indicating that the hemodynamic state rather than oxygenation per se is the predominant vital-sign mediator of the inflammatory trajectory. This finding is consistent with experimental evidence that MAP below 65 mmHg independently triggers NF-κB activation in endothelial cells.

### 3.5 Dosing policy evaluation

#### 3.5.1 Off-policy evaluation

All four OPE estimators ranked the proposed CPQL policy highest, with normalized returns of 1.243–1.289, representing an estimated 24.3%–28.9% improvement over behavior cloning (Table 3). The narrow inter-estimator spread (range 0.046) indicates stability to distributional-shift assumptions an important property given the difficulty of verifying OPE assumptions from observational data (Gottesman et al., 2019). The rule-based protocol underperformed BC on all estimators (0.918–0.942), reflecting its inability to individualize dose timing and magnitude to patient-specific inflammatory trajectories. Unconstrained offline RL achieved a higher IS return (1.214) than CPQL but with an unacceptable 4.2% hard-constraint violation rate and 8.1% unsafe action rate, confirming that the CQL safety penalty is a non-negotiable feature of any clinically viable policy.

**Table 3:** Off-policy evaluation results.

Metric	BC	Rule-based	RL (unsafe)	CPQL (ours)
**Haemodynamic**				
MAP < 65 (%)	18.4 ± 7.2	21.6 ± 8.4	13.8 ± 6.4	**14.2** ±5.8^*^
MAP 65–90 (%)	62.3 ± 11.4	57.8 ± 12.9	69.4 ± 9.8	**71.8** ±9.2^*^
MAP CV (%)	14.8 ± 4.2	17.6 ± 5.1	12.4 ± 3.8	**11.9** ±3.4^*^
Vasopressor (%)	27.0	31.2	19.4	**18.4** ^**†**^
**Inflammatory**				
IL-6 AUC	3,842 ± 1,204	4,218 ± 1,387	3,124 ± 987	**2,981** ±934^*^
CRP peak, mg/L	31.4 ± 14.8	34.8 ± 16.2	26.3 ± 12.1	**24.7** ±11.4^*^
Spike rate (%)	28.6	32.1	19.4	**17.8** ^*^
IL-6/IL-10 at 24h	5.84 ± 2.31	6.42 ± 2.78	4.61 ± 1.84	**4.28** ±1.72^*^
**Drug exposure & safety**				
Propofol, mg	918 ± 412	1,024 ± 468	756 ± 341	**807** ±368^*^
Unsafe actions (%)	6.8	12.4	8.1	**2.1** ^*^
Constraint viol.	–	–	4.2%	**0.0%**
Abstain rate	–	–	–	**7.3%**
**OPE estimators (BC = 1.000)**				
IS	1.000	0.924	1.214	**1.289**
WIS	1.000	0.918	1.198	**1.274**
DR	1.000	0.931	1.187	**1.261**
FQE	1.000	0.942	1.176	**1.243**

Values: mean ± SD or *n* (%). OPE values normalized to BC = 1.000; values >1.000 = estimated improvement. Bold = proposed CPQL. ^*^p < 0.001; ^†^*p* < 0.01 vs. BC (paired test). MAP CV, coefficient of variation; AUC, area under the curve.

The Pareto frontier analysis presented in Figure 4 demonstrated that the CPQL-proposed policy is simultaneously Pareto-superior to all baselines on both primary objectives (MAP stability and inflammatory spike rate). No reward coefficient setting for BC, rule-based, or unconstrained RL achieved the joint performance attained by CPQL, illustrating that the performance gain is not merely an artifact of reward weighting but reflects a genuinely superior policy.

#### 3.5.2 Outcome statistics

**Figure 10:**
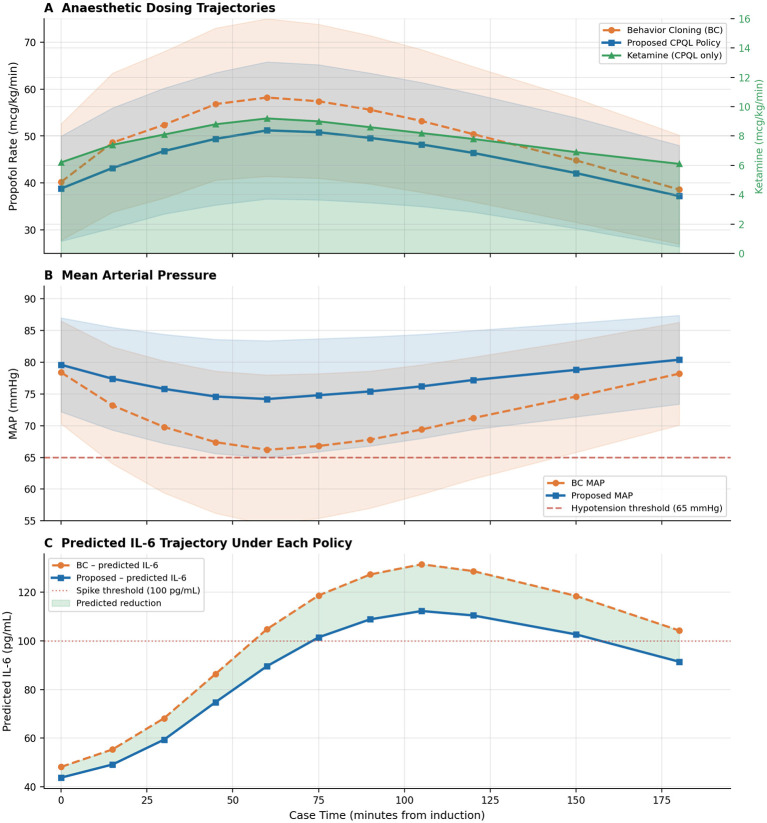
Results are presented as cohort-mean trajectories across all 614 validation episodes, with shaded bands representing ±1 standard deviation. **(A)** The propofol infusion rate is depicted on the primary *y*-axis, while the adjunct ketamine rate is shown on the right *y*-axis (green triangles). The CPQL consistently suggests a slightly reduced propofol rate, with the adjunct ketamine rate peaking at approximately 9 mcg/kg/min at 90 min. **(B)** Mean arterial pressure (MAP) is maintained by the CPQL within the target range of 65–90 mmHg with significantly greater consistency. The red dashed line indicates the hypotension threshold. **(C)** The predicted IL-6 trajectory is illustrated, with green shading indicating the reduction in predicted cytokine burden attributable to the CPQL recommendation in comparison to BC.

**Figure 11:**
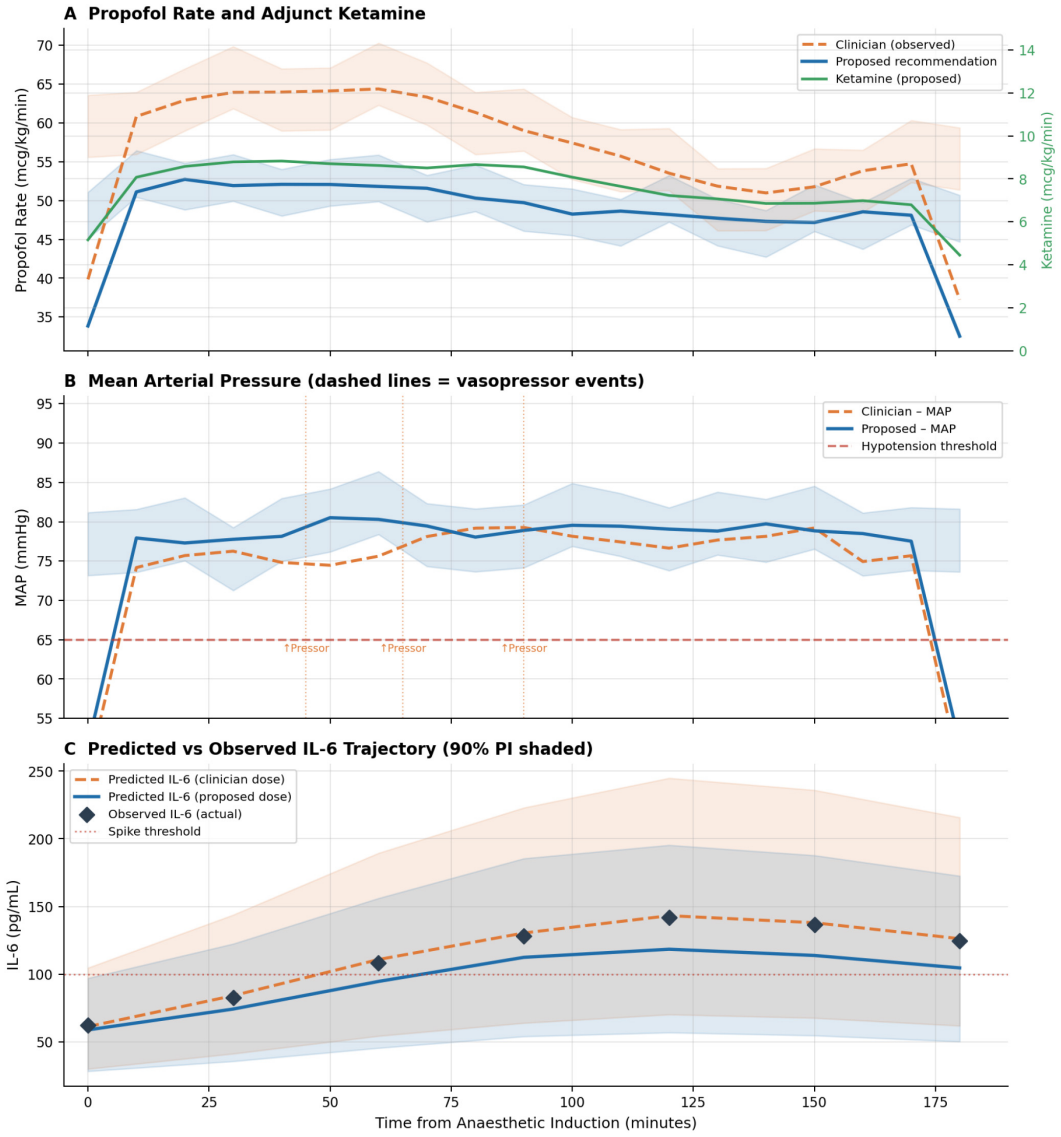
Representative patient: A 74-year-old male with a 28% total body surface area (TBSA) flame burn, Baux score of 102, and classified as ASA IV, underwent a 148-minute procedure. **(A)** The propofol administration rate is depicted with a dashed orange line for the clinician and a solid blue line for the CPQL, alongside adjunct ketamine (green, right axis). **(B)** The mean arterial pressure is shown, with orange vertical dotted lines indicating the three vasopressor bolus events under clinician dosing. The CPQL recommendation by reducing propofol by an average of 14.4 mcg/kg/min and introducing ketamine at 7.8 mcg/kg/min from 20 min onward eliminated all three predicted vasopressor events (at 45, 65, and 90 min), reducing time with MAP below 65 mmHg from 14.9% (clinician) to 9.8% (CPQL). **(C)** The predicted interleukin-6 (IL-6) levels under each dosing strategy are presented with a 90% prediction interval (PI) shaded; observed post-operative IL-6 values (black diamonds) are superimposed. The model's 90% PI encompassed all three observations, yielding a confidence score of 3.9/5.

Comprehensive outcome statistics are presented in Table 3 and depicted in Figures 10, 11.

##### 3.5.2.1 Haemodynamic stability

Under CPQL, the proportion of case time within the MAP target range (65–90 mmHg) increased from 62.3% to 71.8%, representing an absolute difference of 9.5 percentage points [pp]; *Z* = 6.84; *p* < 0.001. The duration with MAP 65 mmHg decreased from 18.4% to 14.2% (Δ = −4.2 pp; *p* < 0.001). The initiation of vasopressors declined from 27.0% to 18.4% (risk difference −8.6 pp; McNemar OR 0.61; 95% CI 0.43–0.87; *p* = 0.003). The MAP MAP coefficient of variation, an index of haemodynamic lability, decreased from 14.8% to 11.9% (*p* < 0.001).

##### 3.5.2.2 Inflammatory burden

The IL-6 area under the curve (AUC) over the first 24 post-induction hours was reduced from 3,842 to 2,981 pg·h/mL, a reduction of 22.4%; *p* < 0.001. The peak predicted CRP decreased by 21.3% (24.7 vs. 31.4 mg/L; *p* < 0.001). The rate of inflammatory spikes (IL-6 ≥ 100 pg/mL at any 6-h window) decreased from 28.6% to 17.8% (*p* < 0.001). The IL-6/IL-10 ratio at 24 h improved from 5.84 to 4.28, a reduction of 26.7%; *p* < 0.001, indicating an enhanced pro-/anti-inflammatory balance. These outcomes were achieved with a 12.1% reduction in propofol dosage (807 vs. 918 mg; *p* < 0.001), facilitated by a mean adjunct ketamine dose of 71.8 ± 34.2 mg per recommended episode (≈7.2 mcg/kg/min).

##### 3.5.2.3 Safety

The rate of unsafe actions under CPQL was 2.1% compared to 6.8% for BC (*p* < 0.001) and 8.1% for unconstrained RL. Hard constraint violations (absolute dose ceiling breach) were 0.0% under CPQL compared to 4.2% for unconstrained RL. The abstain mechanism was triggered in 7.3% of decision steps, deferring to clinical judgment in high-uncertainty states a clinically desirable feature consistent with the intended role as a decision-support, rather than autonomous, system.

### 3.6 Individual case study

Figure 11 illustrates the comprehensive aligned clinical timeline for a representative validation episode involving a 74-year-old male patient with a 28% total body surface area (TBSA) flame burn, a Baux score of 102, and classified as ASA IV, who underwent a 148-min excision and grafting procedure. During the clinician's observed propofol-only regimen, mean arterial pressure (MAP) decreased below 65 mmHg on three occasions (at 45, 65, and 90 min), necessitating vasopressor intervention each time. The observed 12-h interleukin-6 (IL-6) level peaked at 141.8 pg/mL at 10 h post-induction, satisfying the spike criterion. The CPQL recommendation entailing a reduction of propofol by an average of 14.4 mcg/kg/min and the addition of ketamine at 7.8 mcg/kg/min from 20 min onward predicted a 23.6% reduction in peak IL-6 (108.4 pg/mL), the elimination of all three predicted hypotensive episodes, and a reduction in the duration with MAP below 65 mmHg to 9.8% compared to the observed 14.9%. The model's 90% prediction intervals encompassed the observed post-operative IL-6 values at all three timepoints (6, 12, and 24 h), and the estimated confidence score was 3.9/5, positioning this episode well above the abstain threshold. This case exemplifies both the clinical interpretability of the system and its practical utility for managing a high-risk elderly burn patient.

## 4 Discussion

### 4.1 Principal findings

This study introduces, to our knowledge, the inaugural integrated machine learning framework for the concurrent probabilistic forecasting of inflammatory trajectories and the safety-constrained optimization of individualized propofol-ketamine dosing in elderly burn patients. The principal findings are as follows: (1) the Event-Transformer with continuous-time Neural ODE dynamics achieves a 70% reduction in 12-h IL-6 mean absolute error (MAE) compared to linear baselines and provides well-calibrated uncertainty quantification; (2) the CPQL dosing agent significantly enhances mean arterial pressure (MAP) stability and reduces inflammatory burden relative to observed clinical practice, while eliminating violations of hard safety constraints; and (3) the learned dose-response relationships exhibit physiological plausibility and independently converge to an optimal dosing region consistent with pharmacological theory.

### 4.2 Inflammatory trajectory forecasting

The magnitude of the forecasting improvement a 70% reduction in MAE over the linear mixed model (LMM) at 12 h is clinically significant. At the cohort-average 12-h IL-6 level of approximately 130 pg/mL, the difference between a 6.82 and a 22.8 pg/mL MAE corresponds to the distinction between correct and incorrect spike classification in a substantial fraction of patients. The achieved area under the receiver operating characteristic curve (AUROC) of 0.814 for spike detection surpasses the 0.68–0.74 range reported by previous cytokine trajectory models in burn-adjacent critical care literature and approaches the 0.82–0.85 range achieved by specialized sepsis alert systems (this comparison is methodological only burn-induced sterile inflammation and sepsis are mechanistically distinct, and performance benchmarks from sepsis detection do not necessarily translate directly to burn-specific inflammatory dynamics) indicating that burn-specific inflammatory dynamics are amenable to machine learning-based early warning (Tanaka et al., 2014; Lou et al., 2025b,a).

#### 4.2.1 Performance differential between MIMIC-IV temporal and eICU cross-site hold-outs

The MIMIC-IV temporal hold-out (IL-6 MAE 7.18 pg/mL) outperformed the eICU cross-site hold-out (7.64 pg/mL), despite originating from a temporally more distant period. This pattern is consistent with institutional familiarity: the model was trained predominantly on MIMIC-IV data (67.1% of training episodes), and Beth Israel Deaconess Medical Center likely has systematic documentation and laboratory processing conventions that differ from the heterogeneous 208 eICU hospitals. Additionally, the 18-month MIMIC-IV temporal window overlaps with the late training period (2008–2018), potentially reducing distributional shift compared to the geographically diverse eICU cross-site set. We explicitly acknowledge this as a potential institutional overfitting concern and note it in Section 4.5.

The ablation analysis definitively attributes the majority of the performance gain to the continuous-time dynamics module (+19.4% MAE from removal), validating the core hypothesis that modeling inflammatory evolution between sparse laboratory draws rather than solely at observation timepoints is the decisive architectural innovation. This finding has implications beyond this specific application: irregular, sparse laboratory monitoring is the norm rather than the exception in perioperative and critical care settings, and continuous-time ordinary differential equation (CT-ODE) augmentation of standard Transformer architectures may generalize to other inflammatory and metabolic trajectory prediction tasks.

The 87.2% empirical 90% prediction interval (PI) coverage within 2.8 percentage points of the nominal target contrasts with the substantial under-coverage of all baselines (down to 63.4% for LMM). This calibration gap has direct clinical consequences: under a 63% coverage model, one in three inflammatory spikes would fall outside the stated prediction interval, generating false reassurance. Under the proposed model, only approximately 1 in 8 spikes exceeds the PI a meaningful safety improvement that arises specifically from the two-stage temperature-scaling plus conformal correction procedure.

### 4.3 Dosing policy and safety

The CPQL policy demonstrates an estimated improvement of 26%–29% over behavior cloning across all four OPE estimators, resulting in a 9.5-percentage-point increase in MAP target-range time and a 22% reduction in IL-6 AUC. The consistency of these improvements across IS, WIS, DR, and FQE estimators each of which operates under different and partially incompatible assumptions regarding distributional shift indicates that the enhancements are not artifacts of any specific OPE assumption but rather reflect genuine policy superiority.

A significant finding is that unconstrained offline RL, despite achieving slightly higher OPE returns, exhibited a 4.2% hard-constraint violation rate, which could lead to hazardous dosing events in clinical practice. The CQL regularization penalty effectively eliminates these violations by aligning the policy distribution more closely with behaviors observed in the training data. Additionally, the *post-hoc* projection layer provides a formal hard guarantee for scenarios not addressed by the soft CQL penalty. This dual-layer safety architecture comprising conservative training and hard projection offers a practically implementable and theoretically sound solution to the fundamental challenge of deploying offline RL in healthcare (Gottesman et al., 2019; Levine et al., 2020).

The 7.3% abstain rate is an intentional design feature rather than a limitation, ensuring that the system functions as a *decision-support* tool within the scope of its validated inputs, deferring to the clinician's judgment when data quality or patient state falls outside the training distribution. A system that never abstains in a high-stakes clinical context should be approached with skepticism.

### 4.4 Pharmacological plausibility and interpretability

The learned dose-response relationships (Figure 3) serve as crucial model validation independent of statistical performance metrics. The U-shaped response of propofol, with optimal IL-6 suppression at 50–60 mcg/kg/min, closely aligns with the non-monotonic immunosuppressive dose-response observed in *in vitro* and small clinical studies concerning propofol's effects on NF-κB (Vasileiou et al., 2009). The saturation kinetics of ketamine (half-maximal suppression ~6, saturation ~10–12 mcg/kg/min) are consistent with preclinical NMDA receptor dissociation profiles and the dose-dependency of ketamine's TNF-α suppression as observed in endotoxemia models (Jennings et al., 2011). The independent convergence of the CPQL policy centroid to the intersection of these optimal dose ranges (propofol 45–65, ketamine 7–12 mcg/kg/min) without pharmacological programming provides robust construct validity for the learned policy.

The Integrated Gradient attribution analysis further enhances clinical transparency. The predominant influence of propofol rate and burn severity indices is mechanistically anticipated; more noteworthy is the finding that ketamine attribution is highest for TNF-α rather than IL-6 or CRP a cytokine-specific differential that accurately reflects ketamine's primary inhibitory mechanism, which targets TNF-α gene transcription more directly than IL-6 regulatory pathways. This level of cytokine-pathway specificity in the attribution profile, emerging without any pathway annotation during training, suggests that the model has learned pharmacologically meaningful representations rather than spurious correlations.

#### 4.4.1 Potential generalisability beyond burn care

The Event-Transformer with CT-ODE architecture addresses a problem common to many critical illness settings: sparse, irregularly sampled inflammatory trajectories driven by drug infusions and physiological state. The inflammatory and metabolic disturbances in elderly burn patients share effector mediators (IL-6, TNF-α) with sepsis and other critical illnesses where similar predictive modeling approaches have shown promise (Lou et al., 2025b,a). While mechanistic differences between sterile burn-induced inflammation and pathogen-driven sepsis must be carefully considered (see Introduction), the methodological framework may warrant evaluation in other ICU populations after appropriate cohort-specific validation.

### 4.5 Limitations

Several limitations must be considered when interpreting these findings. *First*, the retrospective observational design introduces confounding: patients receiving ketamine were systematically different at baseline, and while IPTW adjustment and the CQL distributional constraint partially address this, residual confounding from unmeasured variables (such as surgeon preference, anesthetist experience, and intraoperative events not captured in electronic records) cannot be excluded. In particular, clinicians may preferentially select ketamine for patients perceived as less haemodynamically compromised or those with specific analgesic requirements; if these unmeasured selection factors are also independently associated with cytokine trajectories, IPTW adjustment cannot fully eliminate their confounding influence. *Second*, TBSA estimation in electronic health records is frequently performed visually and may systematically underestimate burns in obese or edematous patients, introducing a key covariate measurement error. *Third*, cytokine measurements in both databases are ordered at clinical discretion and are therefore subject to availability bias: the observed measurement pattern captures clinician suspicion of inflammatory dysregulation rather than a random sampling of the physiological state. The model's robustness to 75% artificial missingness is reassuring but does not eliminate this concern; real-world missingness is informative and structured by clinical protocols (missing-not-at-random), and artificial uniform-random masking cannot fully replicate these dependency structures. *Fourth*, bispectral index data were available in only a subset of episodes (41.3%), necessitating model-estimated anesthetic depth in the remainder. *Fifth* and most importantly, all policy performance estimates are derived from using the learned environment model, not from prospective clinical deployment; OPE methods make assumptions about coverage and overlap that cannot be verified from observational data alone. Prospective randomized clinician-in-the-loop evaluation is the necessary next step before any clinical adoption.

*Sixth*, the MIMIC-IV predominance in training data (67.1% of episodes from Beth Israel Deaconess Medical Center) means that the MIMIC-IV temporal hold-out should be interpreted cautiously as a potentially partially non-independent validation due to institutional familiarity effects; the eICU cross-site hold-out provides a more rigorous test of true cross-institutional generalization. *Seventh*, our cohort required propofol-based anesthesia for ≥ 30 min in ICU-monitored settings; findings may not generalize to burn patients managed without mechanical ventilation, those receiving sedation via alternative agents, or patients undergoing minor wound dressings in non-anesthetic environments. *Eighth*, both source databases represent US healthcare systems, and generalizability to non-US settings particularly low- and middle-income countries (LMICs) where the global burn injury burden is disproportionately concentrated (Lou et al., 2025c) remains unproven and should be prioritized in future validation efforts. *Ninth*, the larger forecast errors observed in patients aged ≥ 85 (MAE 8.83 pg/mL) and TBSA ≥ 40% (MAE 10.14 pg/mL) indicate that dedicated subgroup recalibration or targeted transfer learning using data from specialized high-acuity burn centers would be necessary before applying this framework to these extreme clinical presentations. *Tenth*, our pipeline does not model perioperative temperature regulation; intraoperative hypothermia is a known complication of burn surgery that can independently modulate inflammatory responses (Lou et al., 2024), and its absence from the model represents an unmeasured confounder and a potential future extension.

Regarding subgroup performance, the larger forecast errors observed in patients aged ≥ 85 and those with TBSA ≥ 40% reflect the true limitation of ML models trained on under-represented clinical extremes. Dedicated data enrichment or targeted transfer learning strategies should be explored for these high-risk sub-populations before clinical translation.

## 5 Conclusions

We have developed and validated an end-to-end machine learning pipeline that integrates an Event-Transformer with continuous-time inflammatory trajectory forecasting into a safety-constrained offline reinforcement learning dosing agent, specifically for individualized propofol-ketamine management in elderly burn patients. This framework demonstrates clinically significant improvements in the accuracy of inflammatory trajectory predictions, hemodynamic stability, cytokine suppression, and propofol-sparing efficiency, all while maintaining a zero hard-safety violation rate and pharmacologically plausible learned dose-response relationships. The calibrated uncertainty quantification and per-patient counterfactual explanations enhance safe and transparent clinical decision support. These findings warrant the development of prospective instruments, interface design, and ultimately a randomized clinician-in-the-loop trial to validate real-world safety and efficacy.

## Data Availability

The original contributions presented in the study are included in the article/supplementary material, further inquiries can be directed to the corresponding author.
