# Supplementary material for: Deep learning guided propofol ketamine dosing and inflammation trajectories in elderly burns
Source: Front Comput Neurosci. 2026 May 18;20:1824898. doi: 10.3389/fncom.2026.1824898 (PMC13223136; doi:10.3389/fncom.2026.1824898)
Supplement: Supplementary file 1 [file Data_Sheet_1.pdf]

frontiers

Supplementary Material

SUPPLEMENTARY FIGURES

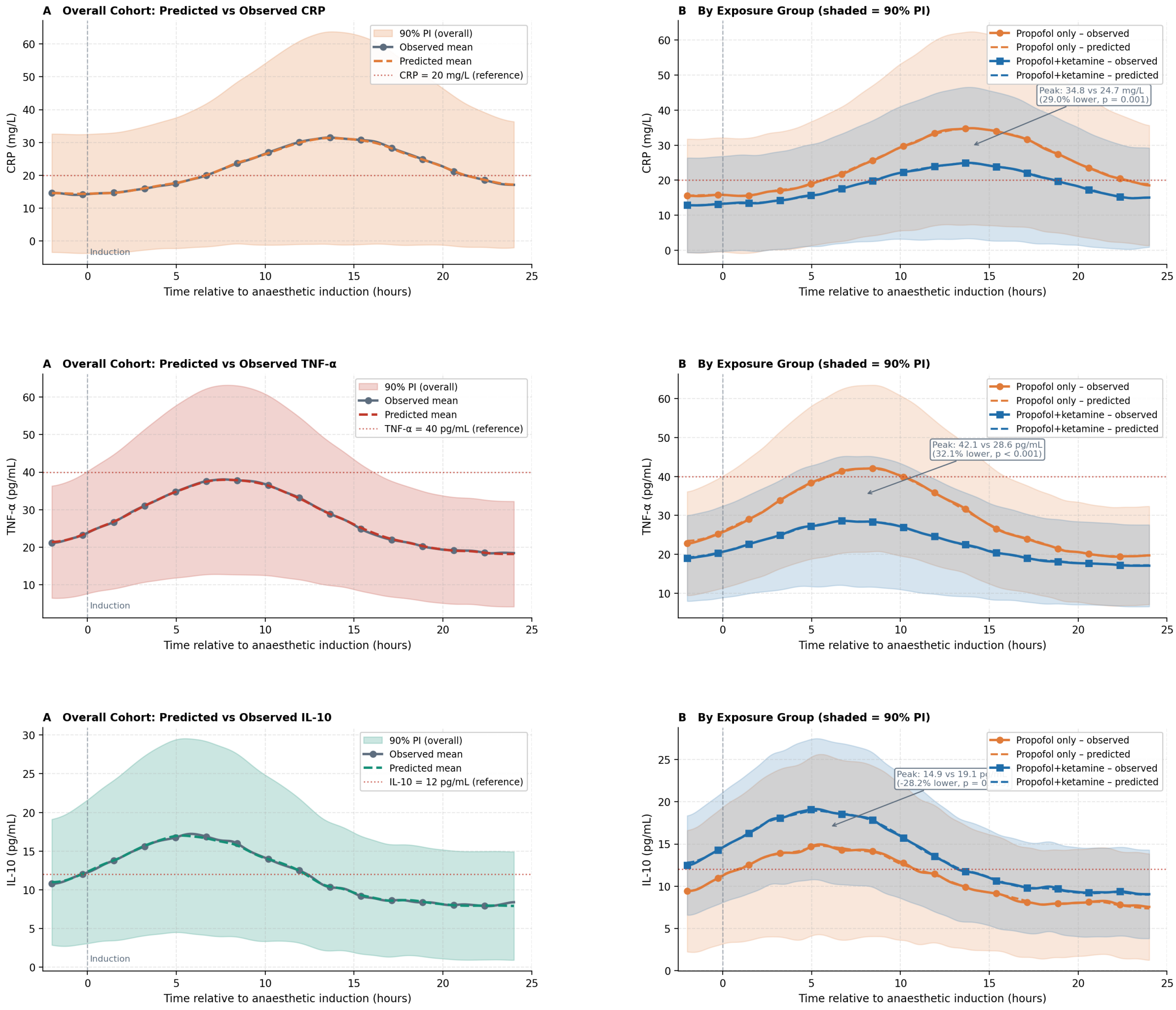

Figure S1. Results from the proposed Event-Transformer with continuous-time ODE dynamics model on the held-out internal validation set (n=123). **Left panels (A)** show overall cohort posterior mean prediction (coloured dashed line) vs observed mean (grey solid line with markers), with 90% prediction interval (shaded band). Dotted red horizontal lines indicate clinical reference thresholds (CRP = 20 mg/L; TNF-α = 40 pg/mL; IL-10 = 12 pg/mL). Vertical grey dashed line marks anaesthetic induction (t=0). **Right panels (B)** stratify results by exposure group: propofol-only arm (orange circles) vs propofol-plus-ketamine arm (blue squares), each with 90% PI shading. Annotations show peak values for each group and the inter-group difference (p-values from paired Wilcoxon test). Consistent with the IL-6 results (main Figure 2), the propofol-plus-ketamine group showed significantly lower peak CRP (34.8 vs 24.7 mg/L, -29.0%, p=0.001), peak TNF-α (42.1 vs 28.6 pg/mL, -32.1%, p<0.001), and a higher peak IL-10 (14.9 vs 19.1 pg/mL, $+28.2\%$, p=0.003), reflecting a more favourable pro-/anti-inflammatory balance under the adjunct ketamine regimen. Forecasting MAE across markers: CRP 1.61 mg/L; TNF-α 3.14 pg/mL; IL-10 2.41 pg/mL (see main Table 2).
